# Single cell transcriptomics reveals spatial and temporal dynamics of gene expression in the developing mouse spinal cord

**DOI:** 10.1242/dev.173807

**Published:** 2019-03-27

**Authors:** Julien Delile, Teresa Rayon, Manuela Melchionda, Amelia Edwards, James Briscoe, Andreas Sagner

**Affiliations:** The Francis Crick Institute, 1 Midland Road, London NW1 1AT, UK

**Keywords:** Spinal cord, Neural development, Neural tube, ScRNA-seq, Single cell transcriptomics

## Abstract

The coordinated spatial and temporal regulation of gene expression in the vertebrate neural tube determines the identity of neural progenitors and the function and physiology of the neurons they generate. Progress has been made deciphering the gene regulatory programmes that are responsible for this process; however, the complexity of the tissue has hampered the systematic analysis of the network and the underlying mechanisms. To address this, we used single cell mRNA sequencing to profile cervical and thoracic regions of the developing mouse neural tube between embryonic days 9.5-13.5. We confirmed that the data accurately recapitulates neural tube development, allowing us to identify new markers for specific progenitor and neuronal populations. In addition, the analysis highlighted a previously underappreciated temporal component to the mechanisms that generate neuronal diversity, and revealed common features in the sequence of transcriptional events that lead to the differentiation of specific neuronal subtypes. Together, the data offer insight into the mechanisms that are responsible for neuronal specification and provide a compendium of gene expression for classifying spinal cord cell types that will support future studies of neural tube development, function and disease.

## Abstract (precis)

**Summary:** Single cell analysis provides an unbiased classification of neural tube cell populations and their associated gene expression profiles, identifying co-regulated genes that mediate a temporal programme of neuronal subtype specification.

## INTRODUCTION

Neuronal circuits in the spinal cord receive and process incoming sensory information from the periphery, and control motor output to coordinate movement and locomotion (Goulding, 2009; Kiehn, 2016). The assembly of these circuits begins on around embryonic day (e)9 in mouse embryos, with the generation of distinct classes of neurons from proliferating progenitor cells that are located at defined positions within the neural tube. This is directed by signals that emanate from the dorsal and ventral poles of the neural tube that partition progenitors into 13 transcriptionally distinct domains that are ordered along the dorsal ventral axis (Alaynick et al., 2011; Briscoe and Small, 2015; Jessell, 2000; Lai et al., 2016; Le Dréau and Martí, 2012). The gene expression programme of each progenitor domain determines the neuronal cell type it generates (Jessell, 2000; Lee and Pfaff, 2001). Neurons differentiate asynchronously from progenitors by undergoing a series of transcriptional changes that converts a proliferative progenitor into specific classes of neurons. Post-mitotic neurons subsequently further diversify into discrete subsets of physiologically distinct neuronal subtypes and commence formation of the circuitry that is characteristic of the spinal cord (Bikoff et al., 2016; Borowska et al., 2013; Goulding, 2009; Hayashi et al., 2018; Kiehn, 2016; Lu et al., 2015). Following the period of neurogenesis, which lasts until ∼e13 in mouse, the remaining undifferentiated progenitors in the neural tube produce glia cells. This is accompanied by specific changes in gene expression in progenitors (Deneen et al., 2006; Kang et al., 2012; Stolt et al., 2003).

Although aspects of the gene regulatory network that controls neural tube patterning and neuronal differentiation have been characterised, there remain substantial gaps in our knowledge. It is unclear whether the complete catalogue of transcriptional regulators that define cell types has been established. Whether there are features in common between the mechanisms that promote the differentiation of distinct neuronal subtypes is not known. Similarly, the alterations in gene expression that accompany temporal changes in progenitor competence and cell type generation are poorly documented. In part, this lack of knowledge is because, until recently, systematic expression profiling studies have been limited to the use of bulk dissected material. The spatial complexity of the neural tube and the lack of developmental synchrony between cells means that bulk studies do not have the resolution or sensitivity to provide detailed insight into gene expression or dynamics in specific cell lineages. Conversely, available single cell expression profiling studies of neural development have either focussed on *in vitro*-derived neural progenitor cells (Briggs et al., 2017; Sagner et al., 2018), or analysed cells from postnatal animals or late-stage embryos (Häring et al., 2018; Rosenberg et al., 2018; Sathyamurthy et al., 2018; Saunders et al., 2018; Zeisel et al., 2018).

To define systematically the complexity of cell types in the developing neural tube and determine the sequence of transcriptional events that is associated with neurogenesis, we performed single cell mRNA-seq analysis. We recovered 21,465 cells from cervical and thoracic regions of the mouse neural tube across five developmental timepoints from e9.5 to e13.5. The data provide an unbiased classification of neural tube cell populations and their associated gene expression profiles. Using this dataset, we inferred the developmental trajectories that lead to distinct neural cell fates and identified cohorts of co-regulated genes that are involved in specific developmental processes. This compendium of gene expression provides a molecular description of neural tube development and suggests testable hypotheses about neural tube development, function and disease.

## RESULTS

### Assignment of transcriptomes to cell identities

**Fig. 1.**
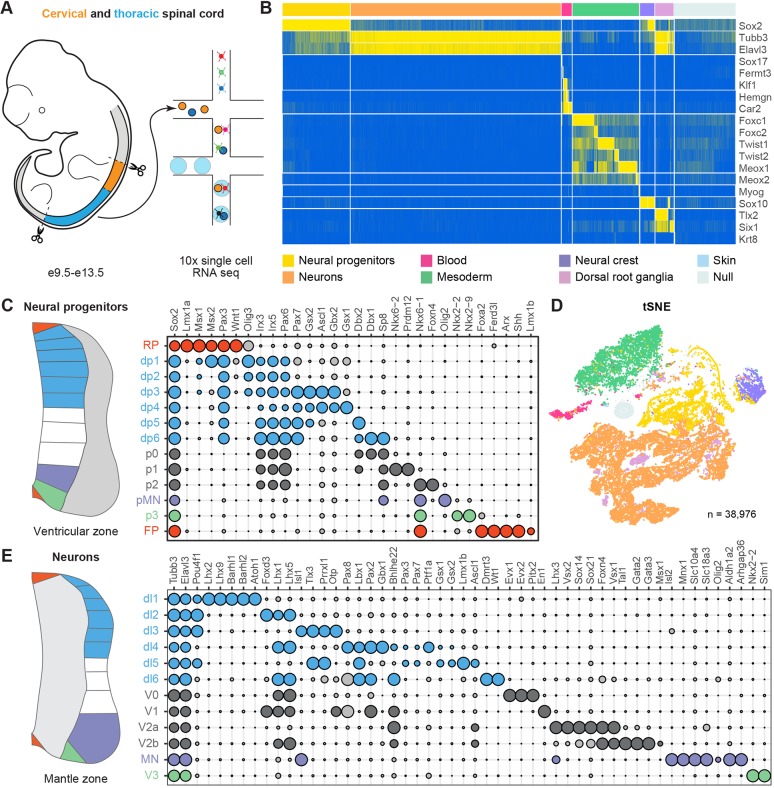
**High throughput scRNA-seq from the developing spinal cord.** (A) Cervical (orange) and thoracic (blue) regions of the spinal cord of mouse embryos from stage e9.5 to e13.5 were dissected, dissociated and sequenced using the 10x Genomics Chromium system. (B) Partitioning of cells to specific tissue types based on the combinatorial expression of known markers. (C,E) Bubble charts that depict the expression of markers used to identify DV domains of progenitors (C) and neuronal classes (E). Circle size indicates normalised gene expression levels. Genes selected for cell categorisation are coloured; grey circles correspond to markers not used for the selection of a specific population. (D) tSNE plot of the entire dataset based on transcriptional similarity using the same markers as B, coloured by assigned cell type. Neural progenitors (yellow) and neurons (orange) were selected for further analysis.

To generate a gene expression atlas of the developing mouse neural tube, we used droplet-based scRNA-seq (10x Genomics Chromium) of microdissected cervical and thoracic regions of the spinal cord from mouse embryos between e9.5 and e13.5 (Fig. 1A). We generated two replicates per timepoint for e9.5, e10.5 and e11.5. To compensate for the increase in size and cell number of the spinal cord, three replicates per timepoint were generated for e12.5 and e13.5. In total, 41,025 cells were sequenced. After applying quality filters, a dataset of 38,976 cells was retained for further analysis (7476 cells from e9.5, 6769 cells from e10.5, 6634 cells from e11.5, 8711 cells from e12.5 and 9386 cells from e13.5) (Fig. S1A). The average number of unique molecular identifiers (UMIs) and detected genes in these cells was similar between all samples analysed (Fig. S1B,C).

We first allocated cells to different tissues based on the combinatorial expression of a curated gene list (Fig. 1B). This allowed the classification of 33,754 cells, 87% of the total cells. The remaining cells did not fall into any of the categories, potentially because of poorly resolved transcriptomes or because these cells were derived from other tissues of the embryo. Further subclustering the unclassified population did not reveal cell populations with spinal cord identity. We also estimated the rate at which the individual transcriptomes might represent more than one cell by assessing the proportion of transcriptomes that displayed a gene expression signature of both neural and mesodermal tissue. This indicated that ∼1% of the transcriptomes were from a mixture of neural and mesodermal cell types (Fig. S1D).

Visualising the resulting dataset with t-distributed stochastic neighbour embedding (tSNE) dimensionality reduction separated cells into multiple groupings that reflected the anticipated cell types (Fig. 1D). Cells from replicate embryos, whether of the same or different sex, tended to intermingle within the embedding, which suggested minimal batch variation between embryos (Fig. S1E,F). By contrast, there were obvious differences in the proportions of cell types from different timepoints. Most neurons were contained in the datasets that were obtained at later developmental stages (Fig. 1D and Fig. S1F), which is consistent with the proportion of neurons increasing in the spinal cord over time (Kicheva et al., 2014). Conversely, most of the neural crest and mesoderm cells originated from e9.5 and e10.5 embryos (Fig. 1D and Fig. S1F). This was because of the difficulty of cleanly dissecting the neural tube without including any accompanying adjacent tissue at these developmental stages.

As it was our aim to construct a spatiotemporal gene expression atlas of the developing spinal cord, we focused on the cells that were identified as spinal cord neural progenitors and neurons. A variety of molecular markers define different domains of progenitors and classes of neurons (Alaynick et al., 2011; Lai et al., 2016). Taking advantage of this, we generated a binary ‘knowledge matrix’ in which each progenitor domain and neuronal class is identified by the expression of a characteristic marker set (see Table S1). Notably, a specific cell type can be defined by more than one combination of marker gene expression patterns, which helps to capture certain subpopulations, such as early neurons that still partially express progenitor markers and have not yet fully activated their neuronal gene expression programmes. We then binarised the expression profiles of the defined marker genes in the transcriptome data and assigned each cell an identity from the knowledge matrix, using the minimal Euclidean distance between each transcriptome and cell type classes. Plotting the resulting average levels of marker gene expression for the assigned cell identities revealed the well-known patterns of progenitor- and neuron-specific gene expression (Fig. 1C,E and Fig. S1G). We therefore conclude that the partitioning algorithm correctly assigns identities to the cells in our dataset.

### Developmental dynamics of cell types

**Fig. 2.**
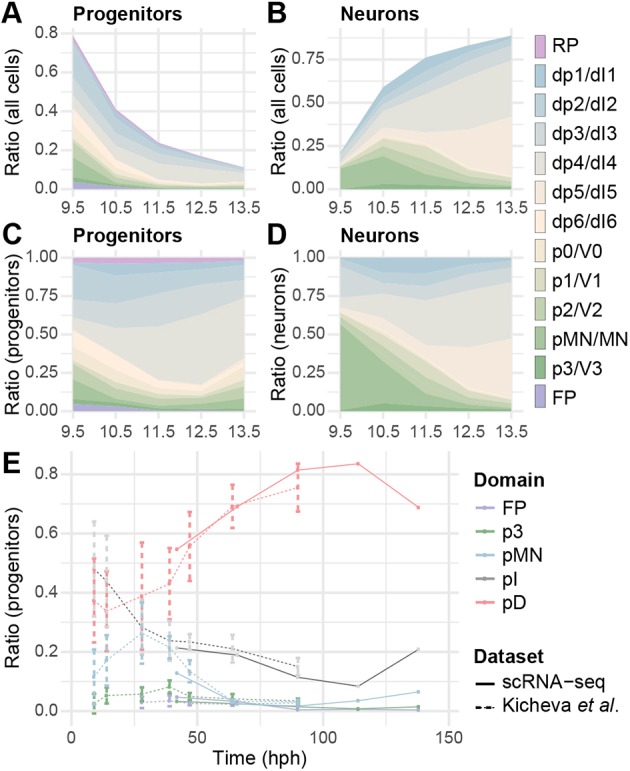
**Dynamics of domain sizes based on the single cell sequencing data.** (A-D) Changes in the fraction of progenitors (A,C) and neurons (B,D) between e9.5 and e13.5. For A and B, the data are normalised to the sum of neurons and progenitors detected at each timepoint. C and D show fractions within progenitors (C) or neurons (D). (E) Comparison of the ratio of progenitors from our dataset (solid lines) with those of Kicheva et al. (2014) (dashed lines). The broader domains pI and pD are composed of p0-p2 and pd1-pd6 progenitors, respectively. hph, hours post headfold.

We hypothesised that sampling several thousand transcriptomes per timepoint would be sufficient to reconstruct the changes in domain sizes during development. Plotting the proportion of progenitors and neurons over time revealed a sharp decrease in the overall proportion of progenitors from >65% of cells at e9.5 to <15% of cells at e13.5 (Fig. 2A). Previous work has indicated that progenitor domain sizes in the spinal cord change over time (Kicheva and Briscoe, 2015; Kicheva et al., 2014). To test whether the proportions of cells that were recovered from the assignment of cell identities accurately recapitulated the growth dynamics of the spinal cord, we compared our results with the quantification in Kicheva et al. (2014). This revealed a close match (Fig. 2E), which allowed us to extend the analysis. Between e11.5 and e12.5, the rate of neurogenesis in the spinal cord appears to slow (Fig. 2A,B); 24 h later, at e13.5, the fraction of progenitors in dorsal domains decreased relative to ventral (Fig. 2C,E). This is in line with the higher rate of neurogenesis in the dorsal spinal cord at e13.5 and the consequent depletion of progenitors (Gross et al., 2002; Lai et al., 2016; Müller et al., 2002).

We performed a similar analysis on the population dynamics of neuronal subtypes (Fig. 2B,D). Motor neurons (MNs) are the most prominent class of neurons at early developmental stages (e9.5 and e10.5), which is consistent with the initially high differentiation rate of MN progenitors (Ericson et al., 1992; Kicheva et al., 2014; Novitch et al., 2001; Sagner et al., 2018) (Fig. 2B,D). At later developmental stages (after e11.5), the proportions of inhibitory dI4 and excitatory dI5 neurons increased markedly (Fig. 2D). This is explained by the expansion of the combined dp4/dp5 progenitor pool in the dorsal spinal cord at these stages and the extended phase of neurogenesis in the dorsal spinal cord that results in the formation of late born dI4 and dI5 neurons (also known as dIL_A_ and dIL_B_) (Gross et al., 2002; Müller et al., 2002). Based on these observations, we conclude that the single cell transcriptomic atlas captures the domain dynamics of progenitor and neuronal populations.

### Prediction of novel gene expression patterns

We sought to identify cell type-specific expression patterns. Here, the challenge was that different cell types are defined by overlapping combinations of gene expression. We constructed the list of all 2^13^ models of combinatorial patterns in progenitor domains (8192 combinations from 13 progenitor domains, including floor plate and roof plate) and we used these to identify genes with differential expression patterns. For each gene, the best-fit model was selected and, after filtering by fold-change and significance level, we obtained a list of 102 combinations (Fig. S2). The same procedure was then applied to the 12 neuronal populations (V3, MN, V2a, V2b, V1, V0, dl6, dl5, dl4, dl3, dl2, dl1) (Fig. S3). From the 4096 combinatorial models that were obtained, we predicted 147 distinct patterns. We applied a conservative *P*-value cutoff of 10^−9^ to obtain a manageable list of candidates from the differential gene expression analysis; lowering this cutoff will extend the list of candidates to pursue in the future.

**Fig. 3.**
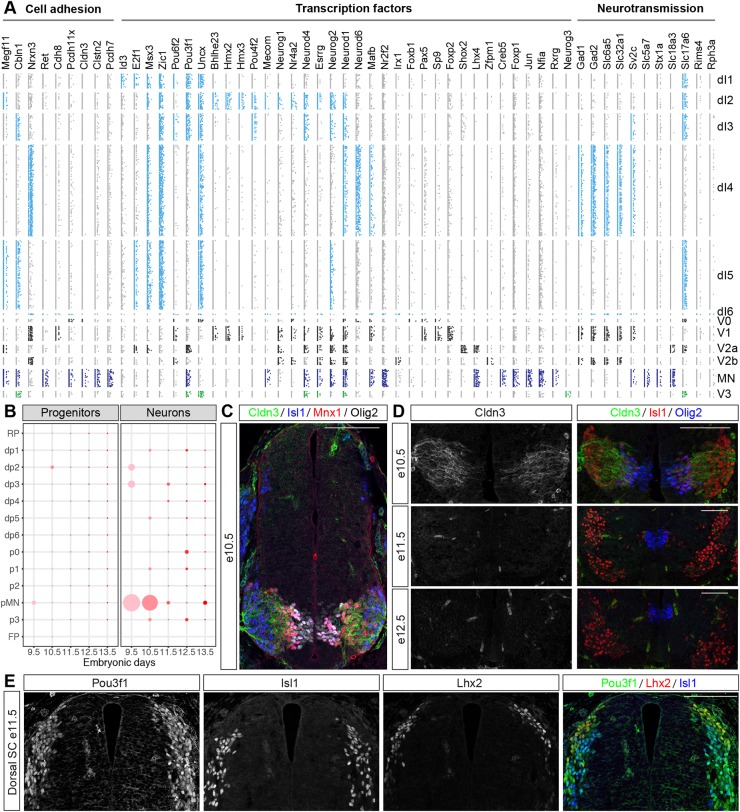
**Spatial and temporal patterns of gene expression in neural progenitors and neurons.** (A) Identification of differentially expressed genes that encode cell adhesion molecules, TFs, and proteins that are involved in neurotransmission. Genes used in the initial partitioning of cell types are not shown. (B) Spatial and temporal expression of Cldn3 in neural progenitors and neurons in the dataset. Cldn3 is specifically expressed in MNs until e10.5. (C) Immunostaining at e10.5 for Cldn3 (green), Isl1 (blue), Mnx1 (red) and Olig2 (grey). (D) Cldn3 expression is specific to MNs at e10.5, and lost in MNs at e11.5 and e12.5. (E) Pou3f1 is expressed in dorsal d1-3 neurons at e11.5. dI1 neurons are labelled by Lhx2 expression and dI3 neurons by Isl1 expression. Scale bars: 100 µm.

For further analysis, we first focused on genes encoding proteins that are involved in neurotransmitter biogenesis and release (Fig. 3A). These identified the three main types of spinal cord neurons: cholinergic MNs, inhibitory GABAergic and glycinergic interneurons, and excitatory glutamatergic interneurons. These could be distinguished based on the expression of metabolic enzymes that are necessary for neurotransmitter production, e.g. glutamatedecarboxylases Gad1 and Gad2, and specific vesicular transporters e.g. Slc18a3 and Slc5a7 (vAcht and high-affinity choline transporter, respectively, in MNs), Slc32a1 and Slc6a5 (vIAAT and Glycine Transporter 2 [GlyT2], respectively, in inhibitory interneurons) and Slc17a6 (vGlut2 in excitatory neurons). The analysis correctly predicted expression in the different neuronal populations: cholinergic MNs; inhibitory GABAergic and glycinergic dI4, V1 and V2b interneurons; and excitatory glutamatergic dI1, dI2, dI3, dI5, V2a, and V3 interneurons (Alaynick et al., 2011; Lai et al., 2016; Lu et al., 2015).

We next examined adhesion molecules (Fig. 3A). In the spinal cord, differential cell adhesion is essential for the clustering of MNs into distinct pools (Demireva et al., 2011; Price et al., 2002) and differential adhesion mediated by neurexins and cerebellins is required for synapse formation and function (Südhof, 2017; Uemura et al., 2010). Interrogation of the data recovered nine differentially expressed cell adhesion molecules (Cldn3, Cbln1, Cdh8, Pcdh11x, Clstn2, Pcdh7, Megf11, Nrxn3 and Ret). Consistent with previous work, the analysis correctly predicted that Pcdh7, Megf11 and Ret are expressed in MNs (Catela et al., 2016; Hanley et al., 2016; Lin et al., 2012). We also found Nrxn3 to be specific for inhibitory neurons in the dI4, dI6, V1 and V2b domains. This pattern was anticorrelated with the expression pattern of Cbln1, which was expressed in excitatory dI3, dI5 and V3 neurons and MNs. Further investigation identified Nrxn1 as specifically expressed in excitatory neurons (dl1, dl2, dl3 and dl5) (data not shown). This raises the possibility of a molecular adhesion code that distinguishes inhibitory and excitatory interneurons in the spinal cord, which might contribute to synaptogenesis during circuit formation.

To validate the inferred gene expression patterns, we focussed on Cldn3. Cldn3 encodes for a tight junction component (Morita et al., 1999) and was predicted to be specifically expressed in MNs before, but not after, e11.5 (Fig. 3B). Assays confirmed this prediction (Fig. 3C,D).

We next turned our attention to transcription factors (TFs) and searched for TFs that were differentially expressed between neuronal subtypes (Fig. 3A). This recovered multiple TFs, many with well-established differential gene expression patterns, for example: Shox2 in V2a neurons (Dougherty et al., 2013; Hayashi et al., 2018); Foxp2 in V1 neurons (Morikawa et al., 2009; Stam et al., 2012); Lhx4 in MNs and V2a neurons (Lee and Pfaff, 2001; Sharma et al., 1998); Neurog3 and Uncx in V3 neurons (Carcagno et al., 2014; Sommer et al., 1996); and high levels of Nr2f2 (also known as COUP-TF2), Nfia, Foxp1 and Creb5 in MNs (Dasen et al., 2008; Glasgow et al., 2017; Hanley et al., 2016; Lutz et al., 1994; Rousso et al., 2008). This analysis also suggested multiple previously unknown expression patterns of TFs, for example: expression of Hmx2 and Hmx3 in V1 and dI2 neurons; Sp9 in V1 neurons; and Bhlhe23 in dI2 neurons (Fig. 3A).

For validation, we assayed Pou3f1 (also known as Oct6 or SCIP). This TF had previously been reported in multiple neuronal subtypes of the spinal cord, e.g. in MNs, V2a and V3 interneurons (Dasen et al., 2005; Francius et al., 2013), which we correctly identified. Our analysis also suggested that Pou3f1 is expressed in dorsal dI1-dI3 neurons (Fig. 3A), which, to our knowledge, had not been described before. Assaying sections of embryonic spinal cord at e11.5 for Pou3f1, the dI1 marker Lhx2 and the dI3 marker Isl1, revealed broad expression of Pou3f1 in dorsal spinal cord neurons and colocalisation between Pou3f1, Lhx2 and Isl1 (Fig. 3E). We also detected Pou3f1-positive cells that did not express either Lhx2 or Isl1; these are presumably dI2 neurons. Thus, the differential gene expression analysis correctly identified the restricted expression patterns of multiple known genes and predicted novel patterns of expression.

### Clustering of neurons predicts novel neuronal subtypes and transcriptional codes

**Fig. 4.**
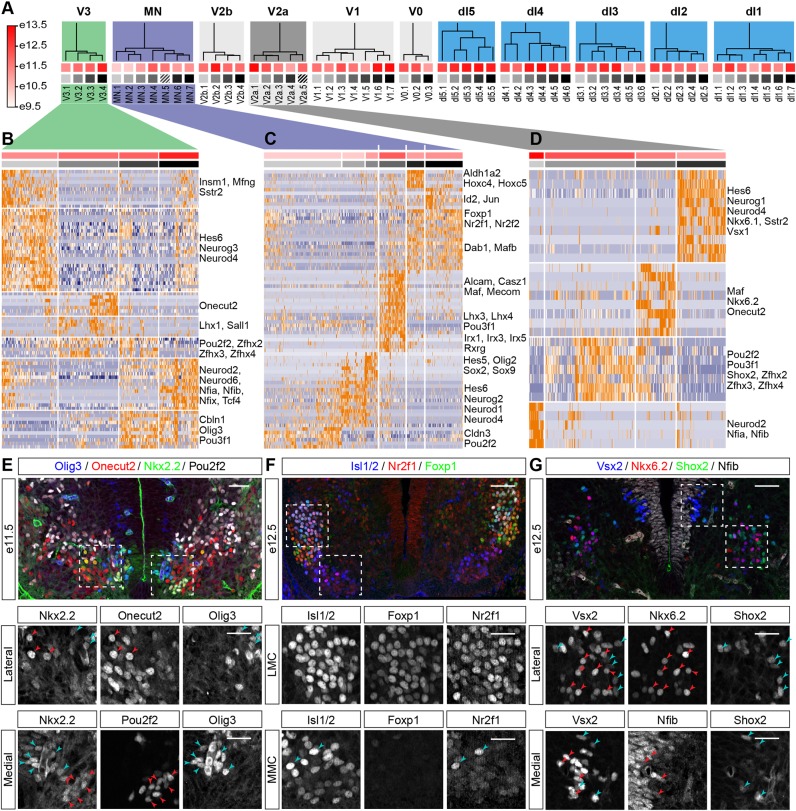
**Hierarchical clustering identifies neuronal subtypes and implicates TFs in determining their identity.** (A) Hierarchical clustering of the cardinal types of spinal cord neurons reveals 59 neuronal subtypes. Dendrograms for each neuronal domain are depicted. Squares under the dendrogram indicate average age (red) and neuronal subtype identity (grey). Colours of squares correspond to those shown on top of the heatmaps in panels B-D and Fig. S4. Striped squares correspond to incorrectly classified cells that were discarded from further analysis. (B-D) Identification of neuronal subtypes by clustering of gene expression profiles in V3 (B), MNs (C) and V2a interneurons (D). Hierarchical clustering was performed using the indicated gene modules (Table S2). A subset of the genes that are included in the modules is indicated on the right-hand side. (E-G) Validation of predicted gene expression patterns obtained from the hierarchical clustering in B-D. Boxed areas are magnified in the middle and bottom rows. Expression of Pou2f2 is detected in Nkx2.2-expressing V3 neurons at e11.5. Pou2f2 expression does not overlap with Onecut2 in more dorsal V3 neurons or Olig3 in V3 neurons abutting the p3 domain (E). Nr2f1 expression is seen in Foxp1-positive LMC neurons at e12.5 (F, middle row). A few Nr2f1-positive cells are also detected in Foxp1-negative MNs within the MMC (F, bottom row). Nkx6.2 expression in lateral Vsx2-expressing V2a neurons does not overlap Shox2 expression at e12.5 (G, middle row). Nfib expression is confined to medial V2a neurons at this stage (G, bottom row). Red arrowheads indicate cells expressing markers in the middle column; blue arrowheads indicate cells expressing markers in the right column. Scale bars: 50 µm; 25 µm in magnified boxed areas.

The 11 neural progenitor domains (p0-p3, pMN, pd1-pd6) generate distinct classes of spinal neurons that further diversify into multiple subpopulations (Alaynick et al., 2011; Bikoff et al., 2016; Francius et al., 2013; Hayashi et al., 2018; Lai et al., 2016; Lu et al., 2015; Sweeney et al., 2018). Gene expression profiles distinguishing subpopulations of neurons have been well documented. To further probe the resolution of the dataset and identify novel components of the gene regulatory network that are involved in neuronal subtype specification, we performed hierarchical clustering independently on each neuronal class (Fig. 4A-D and Fig. S4). We first identified gene modules that consist of genes that demonstrate a concerted pattern of expression in each neuron class. The modules that showed differential expression in a class were used to perform hierarchical clustering and assign distinct subtype identities. This resulted in the identification of 59 subpopulations (Fig. 4A). Of these, initial inspection indicated that only two subpopulations were incorrectly assigned: one group that consisted of poorly characterised neurons lacking any salient transcriptomic feature (clade MN.5) and a second population that expressed late neural progenitor markers Sox9 and Fabp7, which is indicative of progenitor identity (clade V2a.5).

We focused on the identification of subpopulations of neurons in three ventral domains (V2a and V3 interneurons, and MNs) (Fig. 4B-D). V3 interneurons have been previously shown to comprise at least two subtypes with distinct properties and settling positions in the spinal cord (Borowska et al., 2013; Francius et al., 2013; Goulding, 2009; Lu et al., 2015). In e12.5 mice, dorsal V3d neurons express onecut family TFs, whereas ventral V3v neurons express Olig3 (Francius et al., 2013). Hierarchical clustering of V3 neurons revealed at least four different subtypes (Fig. 4B). Clade V3.1 consisted of newly differentiating V3 neurons that expressed the neurogenic markers Neurog3, Hes6 and Neurod4. Clade V3.2 was characterised by expression of Lhx1 and could be further subdivided into a Onecut2-positive and -negative population. Clade V3.3 expressed the TFs Pou2f2, Zfhx2, Zfhx3, and Zfhx4, whereas cells in clade V3.4 expressed Neurod2, Neurod6 and Nfia/b/x. Furthermore, clades V3.3 and V3.4 were characterised by the expression of Pou3f1 and Olig3. Thus, the data correctly identified the different known types of V3 interneurons and predicted the existence of an additional Nfia/b/x- and Neurod2/6-expressing subtype (Fig. 4B).

After their generation, MNs diversify and organise into distinct columns and pools along the anterior-posterior axis of the spinal cord (Francius and Clotman, 2014; Jessell, 2000; Philippidou and Dasen, 2013; Stifani, 2014). Subclustering of the identified MNs revealed six clades (Fig. 4C): Clades MN.2 and MN.3 comprised neurogenic progenitors, because cells in these clades expressed the progenitor markers Sox2, Hes5 and Olig2 (clade MN.3), or Hes6, Neurog2 and Neurod4 (clade MN.2) and did not express substantial levels of the vesicular acetylcholine transporter Slc18a3. The remaining four clades corresponded to more mature neurons. Clade MN.1 comprised early differentiated MNs that were characterised by the expression of the TF Pou2f2 and the tight junction component Cldn3. Median motor column (MMC) neurons and phrenic motor column (PMC) neurons grouped in clade MN.4, which had high levels of Lhx3, Mecom, Pou3f1 and the PMC marker Alcam (Hanley et al., 2016; Stifani, 2014). Two clades (MN.6 and MN.7) of Foxp1-positive cells were identified. Clade MN.6 represented cervical lateral motor column (LMC) neurons, which expressed a gene module containing Aldh1a2 (also known as Raldh2) and the cervical hox genes Hoxc4 and Hoxc5 (Fig. 4C), whereas clade MN.7 comprised thoracic preganglionic motor column (PGC) neurons. Clade MN.6 did not express Hoxc9, which is consistent with the limb level location of the LMC. By contrast, cells in clade MN.7 expressed higher levels of Hoxc9, which is characteristic of the thoracic regions (data not shown).

V2a interneurons have recently been shown to consist of two major subgroups that have different settling positions: a medial subgroup that expresses Nfib and Neurod2, and a lateral subgroup that expresses Shox2 and Zfhx3 (Hayashi et al., 2018). Furthermore, the lateral subgroup appears to diversify further into subgroups that are labelled by Shox2 and Maf/onecut family TFs (Francius et al., 2013; Harris et al., 2018 preprint). Consistent with this, hierarchical clustering of the single cell transcriptome data revealed four major subtypes of V2a neurons (Fig. 4D). Clade V2a.4 consisted mainly of newly differentiating neurons that expressed the neurogenic V2a markers Neurog1, Vsx1 and Hes6, but did not express the mature V2a marker Vsx2 (also known as Chx10). The three other neuronal subtypes were characterised by the expression of Neurod2, Nfia and Nfib (clade V2a.1); Shox2, Pou3f1, Pou2f2, Zfhx2, Zfhx3 and Zfhx4 (clade V2a.2); and Maf and Onecut2 (clade V2a.3). Thus, the data correctly predicted the different subtypes of V2a interneurons.

To test the accuracy of the transcriptome data, we assayed three predictions: the expression of Pou2f2 in V3; Nr2f1 (also known as COUP-TF1) in MNs; and Nkx6.2 in V2a interneurons (Fig. 4B-D). Our analysis suggested that these TFs are expressed in specific subsets of neurons: Pou2f2 in a group of V3 interneurons that is complementary to the Olig3- and Onecut2-expressing populations; Nr2f1 in Foxp1-positive MNs; and Nkx6.2 in a subpopulation of V2a interneurons that does not overlap with Shox2 and Nfib. Assaying expression in e11.5 and e12.5 cervical spinal cord (Fig. 4E-G) confirmed each of the predictions. We therefore conclude that the spinal cord atlas provides sufficient resolution to detect distinct neuronal subtypes and implicates novel TFs in their specification.

### Modular and temporal specification of neuronal identity

**Fig. 5.**
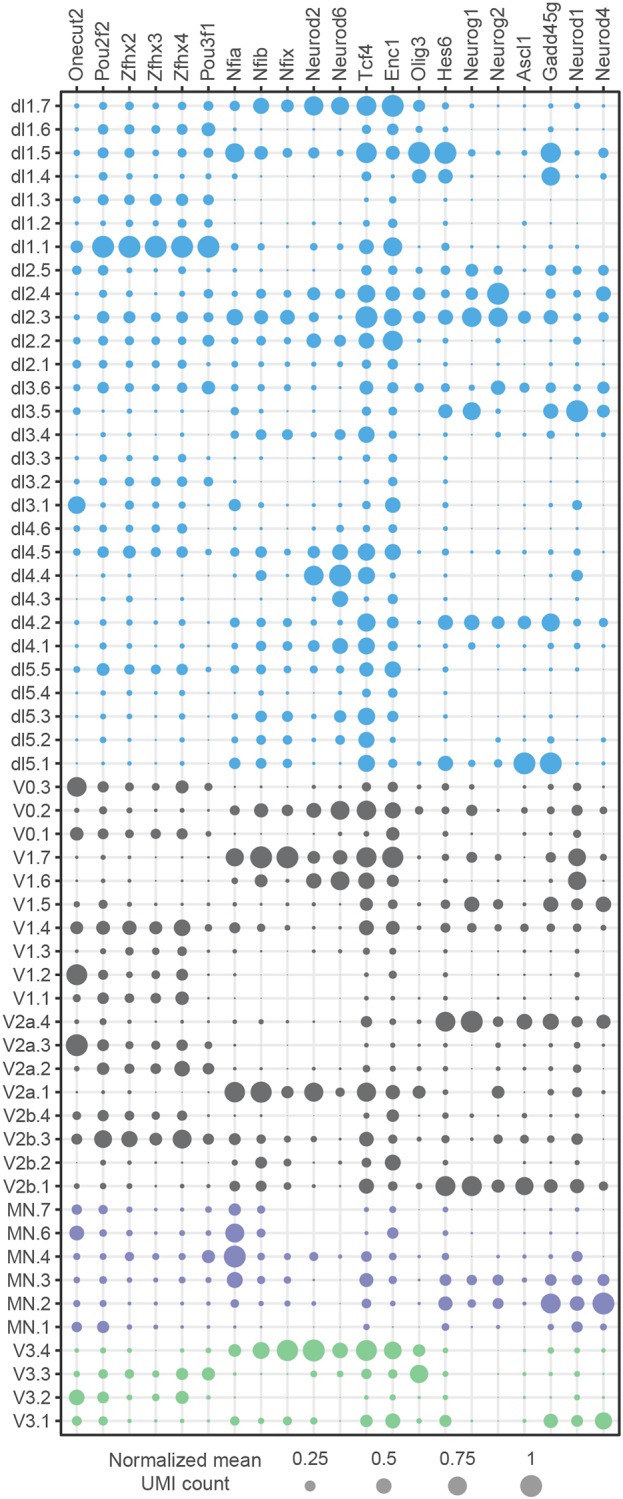
**Subdivision of neuronal classes by a shared set of TFs.** Gene expression profiles of TFs that define subpopulations of neurons in multiple domains. Circle size indicates normalised gene expression levels. Colour indicates domain identities according to Fig. 1E.

Having compiled a gene expression atlas that encompassed multiple cell types and timepoints, we asked whether we could use it to investigate the timing of neuronal subtype generation. We first identified sets of TFs that are used recurrently to define subpopulations of neurons in multiple domains (Fig. 5). Modules containing Pou2f2 and Nfib were expressed in subpopulations of most neuronal classes (Fig. 4B-D and Fig. S4). Furthermore, Pou3f1, Onecut2 and Maf also demarcated subpopulations of multiple neuronal classes, which is consistent with the expression of these TFs in multiple types of neurons (Francius et al., 2013). The composition of the gene modules that contain these TFs was well conserved between neuronal domains. In most cases the Pou2f2 gene module also contained Zfhx2, Zfhx3 and Zfhx4. By contrast, the Nfib gene module often included Nfia, Nfix, Neurod2, Neurod6 and Tcf4. This raised the possibility that similar transcriptional programmes mediate neuronal diversification in each of these domains.

**Fig. 6.**
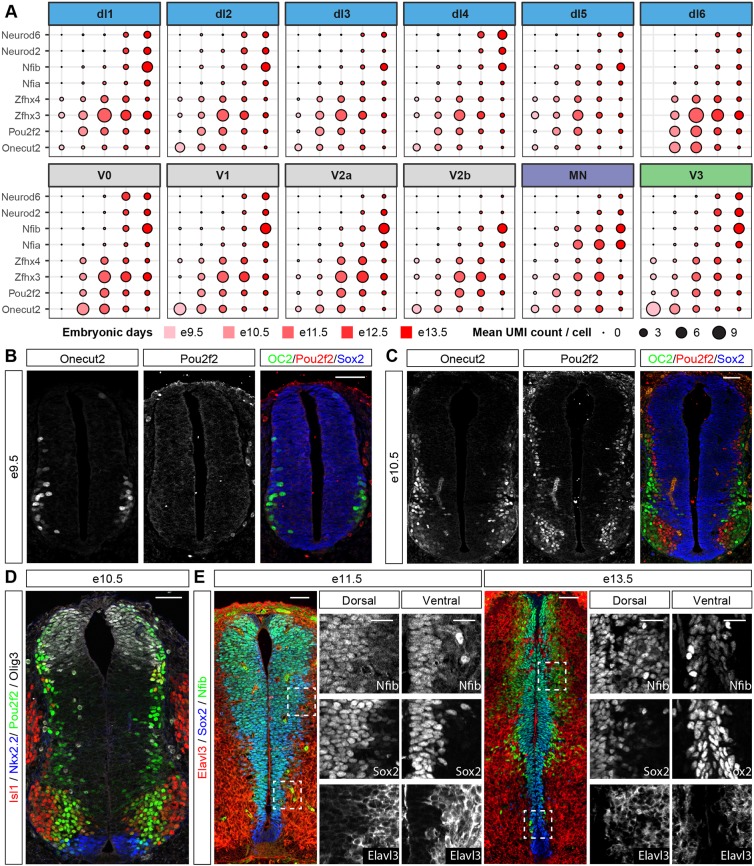
**Temporal stratification of neuronal subtypes by shared sets of TFs.** (A) Temporal expression of a shared set of TFs in different neuronal populations identifies two waves of neurogenesis. The size of the circles indicates the mean expression of genes per stage and domain, and the colour indicates the age of the sample. (B) Onecut2 (OC2), but not Pou2f2, is expressed in neurons at e9.5. (C) Mutually exclusive expression of Onecut2 and Pou2f2 in spinal cord neurons at e10.5. Note that Onecut2-expressing neurons are typically located more laterally than Pou2f2-positive neurons. (D) Widespread expression of Pou2f2 at e10.5 in differentiating neurons close to the ventricular zone. Pou2f2 expression colocalises with Olig3, Nkx2.2 and Isl1. (E) Increased expression of Nfib in differentiating neurons at late developmental stages. Boxed areas are magnified in the middle and right rows for each developmental stage. Nfib is expressed at low levels at e11.5 in progenitors that are labelled with Sox2 and is not detected in neurons. By contrast, at e13.5 Nfib expression is observed in neurons that also express the neuronal marker Elavl3. Scale bars: 50 µm; 25 µm in in magnified boxed areas.

We asked whether specific gene modules were induced at different timepoints in development. Examining the average expression level of Onecut2, Pou2f2, Zfhx3, Zfhx4, Nfia, Nfib, Neurod2 and Neurod6 for each neuronal class between e9.5 and e13.5 revealed a temporal stratification of neuronal subtypes that was conserved between domains (Fig. 6A). The proportion of neurons that expressed Onecut2 and Pou2f2 peaked at early developmental stages (before e11.5), whereas Nfib and Neurod2 expression were induced at e12.5 or e13.5. This is consistent with previous observations: onecut TFs have been shown to be expressed early in V1 and MNs (Roy et al., 2012; Stam et al., 2012) and Nfia/b are induced in MNs at later timepoints (Deneen et al., 2006; Kang et al., 2012). To test directly for the temporal progression between subtypes, we first focussed on Onecut2 and Pou2f2. At e9.5 Onecut2 is expressed in neurons, whereas Pou2f2 was not detected at this stage (Fig. 6B). At e10.5, expression of both markers was mutually exclusive, with Onecut2-positive neurons typically occupying a more lateral position in the spinal cord than Pou2f2 neurons (Fig. 6C). These observations are consistent with the single cell sequencing data and suggest a switch in neuronal subtype identity from Onecut2 to Pou2f2 between e9.5 and e10.5. Similar to Pou2f2 (Fig. 6D), staining for Zfhx3 confirmed its expression in subsets of neurons at e10.5 (Fig. S5A), whereas Nfib and Neurod2 were not expressed in neurons until after e11.5 (Fig. 6E and Fig. S5B). Furthermore, Pou2f2 and Nfib were co-expressed with multiple domain-specific markers at the respective stages (Fig. S5C-E). Based on these observations, we conclude that neuronal subtype diversification in the spinal cord is at least partly driven by transcriptional programmes that are shared between multiple domains and sequentially induced in each of the domains. This suggests a temporal component to the specification of neuronal subtype identity that complements the well described spatial axis of patterning.

### Reconstruction of the gene expression dynamics underlying neurogenesis

**Fig. 7.**
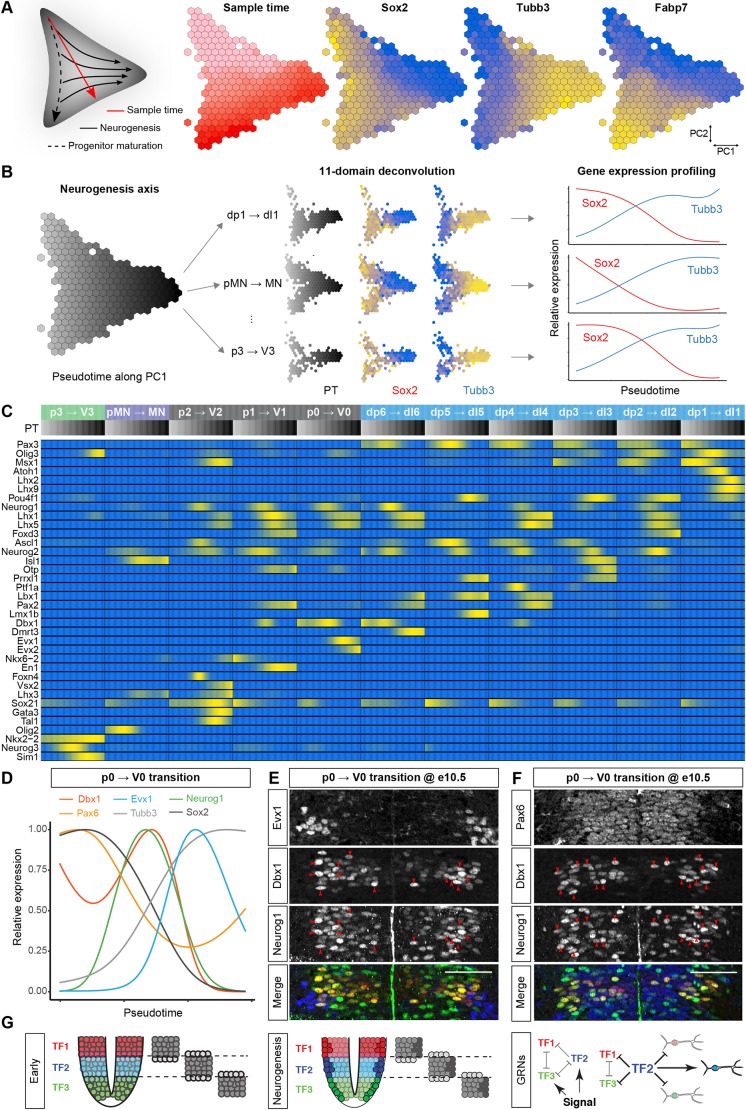
**Pseudotemporal ordering reveals gene expression dynamics during neurogenesis.** (A) PCA projection of all neural cells, shown on a hexagonal heatmap, from a 100-dimensional space that was defined by genes expressed in all DV domains during progenitor maturation and neurogenesis. The schematic on the left depicts progenitor maturation (top to bottom), and neurogenesis (left to right). The hexagonal heatmaps indicate the number of cells from different developmental stages and the expression pattern of the pan-progenitor marker Sox2, the neuronal marker Tubb3, and the gliogenic marker Fabp7 (blue, low; yellow, high). (B) The first principal component of the cell state graph was used to independently reconstruct neurogenesis in each DV domain. Cells allocated to specific DV domains were plotted along the differentiation trajectory, and the expression profile of genes were independently reconstructed. (C) Upregulation of domain-specific TFs coincides with neurogenesis in multiple domains. Heatmap, including the normalised expression pattern (blue, low; yellow, high) of genes that are involved in neurogenesis per domain along the pseudotemporal (PT) axis (grey, early; black, late). (D) Smoothed expression profile of the neurogenic trajectory from p0 to V0 shows a transient upregulation of Dbx1 before neurogenesis that coincides with the maximal expression of Neurog1. (E,F) Upregulation of Dbx1 coincides with Neurog1 expression. Co-expression of Dbx1 and Neurog1 in cells in the differentiation zone of the ventricular layer in the p0 domain at eE10.5 (E). V0 neurons are identified by the expression of Evx1. Although Dbx1 expression is maximal in differentiating progenitors at e10.5, Pax6 expression is homogeneous in all p0 progenitors (F). Red arrowheads indicate cells expressing Neurog1 and high levels of Dbx1. (G) Domain-specific TFs are upregulated before neurogenesis. Initially, domain-specific TFs specify progenitor identity. Upon neurogenesis, domain-specific TF expression is transiently upregulated to reinforce the subtype identity of the differentiating neurons. Scale bars: 50 µm.

Finally, we turned our attention to the changes in gene expression that accompany the transition of progenitors to neurons. Single cell transcriptome analysis enables the reconstruction of gene expression dynamics along differentiation trajectories (Setty et al., 2016; Shin et al., 2015; Trapnell et al., 2014). To this end, we projected cells into a two-dimensional space (Fig. 7A) using principal component analysis (PCA) from a 100-dimensional space that was defined by a set of genes expressed in all dorsal-ventral (DV) domains during progenitor maturation and neurogenesis (Fig. S6A). The first principal component aligned with neurogenesis, which was indicated by the downregulation of Sox2 and upregulation of Tubb3, hence we used PC1 coordinates as a pseudotemporal ordering for neurogenesis. We then reconstructed, independently for each domain, expression profiles of genes along pseudotime (Fig. 7B). This predicted the expression changes in a set of 36 regionally patterned genes that are involved in neurogenesis (Fig. 7C).

Examining gene expression changes as progenitors transitioned to neurons revealed the transient upregulation of several TFs. These included known neurogenic factors, including Atoh1, Neurog1 and Neurog2, and several domain-specific TFs. In line with this, we have recently demonstrated that upregulation of Olig2 coincides with Neurog2 expression and neurogenesis in MN progenitors (Sagner et al., 2018). To test whether similar expression dynamics occur during neuronal differentiation in other progenitor domains, we first examined Olig3, which is expressed in the three most dorsal progenitor domains, dp1-dp3 (Müller et al., 2005). Differentiation of these progenitors into dI1-dI3 neurons depends on the expression of the proneural bHLH proteins: Atoh1 in dp1; Neurog1 in dp2; and Neurog2 in dp2 and dp3 progenitors (Lai et al., 2016). Plotting Olig3 expression dynamics along the differentiation trajectory from dp2 to dI2 neurons or from dp3 to dI3 neurons revealed that the maximal expression of Olig3 coincided with the expression of Neurog1 and Neurog2 (Fig. S6C,D). To test the specificity of the transient upregulation of Olig3, we examined the dynamics of Msx1 and Pax3. In contrast to Olig3, expression of both genes decreased monotonically during differentiation (Fig. S6C,D). Assays of spinal cord sections revealed heterogeneity in Olig3 levels, with higher expression of Olig3 correlating with Neurog1 and Neurog2 expression and low levels of Msx1 (Fig. S6F-H). These observations confirm that Olig3 expression is upregulated at the onset of neurogenesis and validate the predictions that were made by the pseudotemporal ordering.

Similar reconstruction of the p0 to V0 transition predicted that Dbx1 levels increase during neurogenesis (Fig. 7D). Moreover, levels of the p3 marker Nkx2.2 were predicted to increase at the onset of neurogenesis (Fig. S6B). Assays confirmed these predictions: Dbx1 levels were markedly elevated in Neurog1-positive p0 progenitors (Fig. 7E), whereas Nkx2.2 levels were increased immediately adjacent to the p3 progenitor domain in Olig3-positive V3 neurons (Fig. S6E). In both cases, upregulation was specific to the domain-specific TFs Dbx1 and Nkx2.2, but, consistent with the predictions that were made by the pseudotemporal ordering, not seen for the more broadly expressed progenitor TFs Pax6 and Sox2 (Fig. 7F and Fig. S6E).

## DISCUSSION

We have documented the transcriptional signatures of 21,465 cells that were isolated from cervical and thoracic regions of the mouse neural tube during the developmental period in which neuronal subtypes are generated. This sheds light on the changing gene expression profiles that characterise neural tube development and forms the basis of a molecular atlas of the developing mammalian spinal cord.

### An atlas of spinal cord gene expression

The number of cells that are needed to generate a comprehensive atlas of a tissue depends on multiple factors, which include the number of cell types and the molecular differences between the cell types (Shekhar et al., 2016). There is a large diversity of cell types in the spinal cord. More than 50 distinct pools of MNs have been documented at limb levels of the spinal cord (Dasen et al., 2005; Landmesser, 2001; Philippidou and Dasen, 2013). Moreover, the combinatorial expression of 19 TFs have been proposed to generate multiple subpopulations of V1 interneurons (Bikoff et al., 2016; Gabitto et al., 2016; Sweeney et al., 2018). Even though some of this diversity may arise at developmental stages later than those analysed in our study, it is likely that our analysis underestimates the diversity of cell types in the spinal cord. Increasing the number of cells sampled, particularly at e12.5 and e13.5, and improving the sensitivity of the methods to increase the complexity of the analysed transcriptomes might reveal further cell type diversity. In addition, neuronal subtypes vary along the rostral caudal axis of the neural tube (Hayashi et al., 2018; Philippidou and Dasen, 2013; Sweeney et al., 2018). We restricted our analysis to cervical and thoracic regions of the neural tube, and therefore our dataset will not represent cell types that are unique to lumbar and sacral spinal cord. Despite these limitations, we were able to detect all the progenitor populations and major neuronal subtypes that are described in cervical and thoracic regions of the spinal cord. Reassuringly, the proportions of the cell types recovered matched previously determined proportions in the spinal cord (Kicheva et al., 2014), which suggests that the methods did not substantially bias the composition of the transcriptomes recovered. Consistent with this, relatively small subclasses of several of the neuronal subtypes, such as V3, V2a and dI6, and specific subtypes of MNs, including LMC and MMC MNs, were readily detected.

Although much progress has been made to reverse engineer the gene regulatory network that is responsible for pattern formation and cell fate specification in the neural tube (reviewed by Briscoe and Small, 2015; Lai et al., 2016), it remains incomplete. This study makes available detailed information on the transcriptional state of the entire population of neural tube cells and will likely accelerate efforts to map comprehensively the transcriptional network. To assemble the single cell transcriptomic atlas, we took advantage of the extensive prior knowledge of gene expression to analyse and organise the transcriptome data (Fig. 1C,E). This allowed us to expand the molecular description of cell types and define new subdivisions of neurons. Importantly, experimental assays corroborated the transcriptome predictions, which suggests that the atlas is a generally faithful representation of gene expression in the spinal cord. Further mining the dataset will likely implicate additional TFs in the neural tube gene regulatory network and refine the molecular classification of cell types.

### Temporal specification of neuronal subtype identity

The data highlighted a previously underappreciated systematic temporal component to the specification of neuronal subtype identity in the neural tube. Temporal mechanisms, in which a sequence of TFs is expressed in succession to determine distinct neuronal identities, are well established in cortical neurogenesis and *Drosophila* neuroblasts (Holguera and Desplan, 2018). In the spinal cord, the birth date of spinal neurons has been shown to correlate with subtype identity and functional properties in several specific cases (Hayashi et al., 2018; Hollyday and Hamburger, 1977; Sockanathan and Jessell, 1998; Stam et al., 2012; Tripodi et al., 2011). Whether this is a consequence of a global patterning strategy that involves temporally organised changes in gene expression has been unclear. Analysis of the single cell transcriptome dataset revealed a set of gene expression modules that is activated synchronously at characteristic developmental timepoints in multiple neuronal classes. This raises the possibility of a coordinated temporal transcriptional programme that subdivides neuronal classes based on their time of generation. Such a mechanism would operate in parallel to the spatial patterning mechanisms and provide an opportunity to increase the molecular and functional diversity of cell types that are generated in the neural tube.

The coordinated induction of gene modules in multiple neuronal subtypes might result from a global transcriptional change in the neural progenitors from which they are generated. In the spinal cord, a transcriptional cascade of Sox9 and Nfia/b in progenitors underlies the progressive activation of gliogenesis (Deneen et al., 2006; Kang et al., 2012; Stolt et al., 2003). Sox9 expression begins between e9.5 and e10.5, whereas Nfia/b is expressed from e11.5 (Deneen et al., 2006; Stolt et al., 2003). However, the forced expression of these TFs does not repress neurogenesis, and neurons continue to be produced for a considerable period after their expression commences (Deneen et al., 2006). The onset of expression of Sox9 coincides with the switch of V1 neurons from Renshaw cells (onecut-expressing) to Foxp2-expressing neurons (Kang et al., 2012; Stam et al., 2012; Stolt et al., 2003). This raises the possibility that the induction of TFs, such as Sox9 and Nfia/b, changes the competency of neural progenitors to give rise to specific neuronal subtypes and that neural progenitors within a single domain are different over time. In this view, the same mechanism that is responsible for the activation of gliogenesis in neural progenitors serves as a mechanism to generate neuronal diversity in a coordinated manner throughout the spinal cord. Experiments are required to test this and investigate whether similar principles apply to other regions of the nervous system.

Extrinsic signals may contribute to the temporal stratification of neuronal subtypes. Retinoic acid (RA) promotes Renshaw cell identity in V1 neurons (Hoang et al., 2018), and the timepoint of their generation correlates with expression of the RA-synthesising enzyme Aldh1a2 in the adjacent somites (Niederreither et al., 1997). Thus, besides promoting neurogenesis (Novitch et al., 2003), somite-derived RA may be a determinant for early onecut-positive neuronal subtypes. Another candidate for mediating the temporal progression of neuronal subtypes is TGFβ signalling, which has been shown to suppress early born neuronal identities in favour of later born cell types in multiple regions of the nervous system (Dias et al., 2014). Lastly, we observed the expression of FGF ligands in multiple neuronal subtypes, whereas neural progenitors at later stages upregulated FGF receptor3 (Fgfr3) and FGF-binding protein 3 (Fgfbp3) (Gonzalez-Quevedo et al., 2010; Kang et al., 2012). Thus, multiple secreted signalling molecules may determine into which neuronal subtypes progenitors in the spinal cord differentiate at specific timepoints.

### Dynamics of gene expression during neurogenesis

We have previously demonstrated that upregulation of Olig2 precedes MN formation *in vivo* and *in vitro* (Sagner et al., 2018). Pseudotemporal ordering of the progenitor-to-neuron transition for multiple progenitor domains predicted the transient upregulation of domain-specific TFs during neurogenesis (Fig. 7C). We confirmed this experimentally for Olig3 in dp2/3, Dbx1 in p0, and Nkx2.2 before neurogenesis reinforces neuronal specificity during differentiation (Fig. 7G). At early developmental stages, morphogen gradients establish discrete domains of progenitor identities along the DV axis by inducing distinct TFs (Alaynick et al., 2011; Briscoe and Small, 2015; Le Dréau and Martí, 2012). These TFs form a gene regulatory network that establishes progenitor identities by repressing not only adjacent progenitor identities but also a wide range of alternative fates (Kutejova et al., 2016; Nishi et al., 2015). The dynamics of the gene regulatory network result in progenitors undergoing a succession of changes in TF expression during their specification, which is mediated by repressive interactions. The lower levels of domain-specific TFs in progenitors, before neurogenesis, might therefore ensure sensitivity to morphogen inputs and facilitate cell-state transitions in response to morphogen inputs. Such a mechanism, however, could be prone to the generation of mixed neuronal identities as neurogenesis commences (Ericson et al., 1996). The upregulation of the domain-specific TFs during neurogenesis might serve to consolidate the appropriate identity and to prevent the initiation of a mixed neuronal identity during differentiation. Thus, the upregulation of domain-specific TFs during neuronal differentiation could provide a means to enhance the fidelity of spinal cord patterning.

In conclusion, we document the molecular diversity and cellular composition of the developing mouse neural tube. The data allow the identification of genes and regulatory modules that define specific cell types. The analysis suggests a temporal axis that contributes to the neuronal diversity that accompanies the well-characterised spatial patterning of neural progenitors. Together, the data provide new opportunities to understand gene function and to target cells genetically for visualisation or manipulation, and will support efforts to understand the structure and function of the healthy as well as diseased or damaged, spinal cord.

## MATERIALS AND METHODS

### Animal welfare

Animal experiments were performed under UK Home Office project licenses (PD415DD17) within the conditions of the Animal (Scientific Procedures) Act 1986. Outbred UKCrl:CD1 (ICR) (Charles River) mice were used for this study.

### Immunofluorescent staining and recording of spinal cord sections

Mouse spinal cord tissues were fixed in 4% paraformaldehyde (Thermo Fisher Scientific) in PBS, cryoprotected in 15% sucrose, embedded in gelatine, and 14 µm sections taken. A complete list of primary antibodies (including manufacturer and dilutions) is provided in Table S3. Secondary antibodies used throughout this study were raised in donkey (Life Technologies; Jackson Immunoresearch; Sigma-Aldrich; Biotium). Alexa488- and Alexa568-conjugated antibodies were used at 1:1000 dilution, Alexa647-conjugated antibodies were used at 1:500 and CF405M-conjugated secondary antibodies at 1:250.

Images were acquired on a Zeiss Imager.Z2 microscope equipped with an Apotome.2 structured illumination module and a 20× air objective (NA=0.75). Five-phase images were acquired for structured illumination. *Z*-stacks consisted of eight sections separated by 1 µm. Individual optical slices are shown. If required, adjacent images were acquired with 5-10% of overlap and stitching was performed in Fiji using the ‘Grid/Collection stitching’ plugin (Preibisch et al., 2009).

### Sample preparation for single cell RNA sequencing

Outbred CD1 females were timed mated to generate mouse embryos of the specified stages. The morning of the vaginal plug was defined as e0.5. For neural tube dissection, cervical and thoracic sections of single mouse embryos were dissected in Hanks Balanced Solution without calcium and magnesium (HBSS, Life Technologies, 14185045) supplemented with 5% heat-inactivated foetal bovine serum (FBS). The samples were then incubated on FACSmax cell dissociation solution (Amsbio, T200100) with 10× Papain (30 U/mg, Sigma-Aldrich, 10108014001) for 11 min at 37°C to dissociate the cells. To generate a single cell suspension, samples were transferred to HBSS, with 5% FBS, rock inhibitor (10 μM, Stemcell Technologies, Y-27632) and 1× non-essential amino acids (Thermo Fisher Scientific, 11140035), disaggregated through pipetting, and filtered once through 0.35 μm filters and once through 0.20 μm strainers (Miltenyi Biotech, 130-101-812). Quality control was assayed by measuring live cells versus cell death, cell size and number of clumps. Samples with a viability above 65% were used for sequencing, and 10,000 cells per sample were loaded for sequencing.

### Single cell generation, cDNA synthesis and library construction

A suspension of 10,000 single cells was loaded onto the 10x Genomics Single Cell 3′ Chip, and cDNA synthesis and library construction were performed as per the manufacturer's protocol for the Chromium Single Cell 3′ v2 protocol (10x Genomics; PN-120233). cDNA amplification involved 12 PCR cycles.

### Nucleic acid sequencing protocol

Libraries for the samples were multiplexed so that the number of reads matched one lane per sample, and sequenced on an Illumina HiSeq 4000 using 100 bp paired-end runs. Libraries were generated in independent runs for the different samples and for different timepoints.

### Alignment and preparation of scRNA-seq data

Demultiplexing, alignment, filtering, as well as barcode and UMI counting, were performed using Cell Ranger (version 2.2.0, 10x Genomics, under default settings), which uses STAR aligner. The aggregated gene-barcode matrix was normalised by subsampling reads from higher-depth libraries until all samples had an equal number of confidently mapped reads per cell. Further analysis was performed using R.

### Quality filtering

We excluded cells that had more than 6% UMI counts associated with mitochondrial genes and that expressed less than 500 genes.

### Data analysis

We partitioned cells into 13 progenitor and 12 neuronal populations using the following two-step strategy. First, we determined the global cell identities by associating each cell to the closest target population state that was defined by a list of known marker genes (Fig. 1B). The binarised levels were obtained using a threshold of two UMI counts and cell-to-target distances were calculated by Euclidean distance. Second, each progenitor and neuronal population was further partitioned using the same approach using the list of marker genes shown in Fig. 1C,E.

### Combinatorial testing for differential expression

To classify the whole transcriptome by categories of spatial DV patterns, we performed a series of differential expression tests. The analysis was performed independently on progenitors and neurons. In both cases, the procedure was identical: for each gene, 2*^N^*-2 (where *N*=13 for progenitor domains and *N*=12 for neuronal domains, respectively) approximate χ^2^ likelihood-ratio tests were run between a null hypothesis that models gene expression as a function of a single sample (no predictor) versus an alternative hypothesis modelling gene expression as a function of two samples; the ‘positive’ sample being one of the potential population combinations and the ‘negative’ sample the complementary combination. Hence, all combinations were tested. The tests were run using Monocle (version 2.6.4) ‘differentialGeneTest’ function with gene level distributions modelled as negative binomial distributions with fixed variance [option ‘expressionFamily=negbinomial.size()’, as recommended for UMI datasets]. Each gene was then associated with the population combination for which it obtained the highest likelihood. The gene list was trimmed using significance (*P*<10^−9^) and fold-change (log2-fc>2) cutoffs. We also ensured that the remaining genes had a minimal average level of expression (0.2 UMI) and a ratio of expressed cells (10% in progenitors, 8% in neurons) in the positive samples.

The gene sets highlighted in Fig. 3A were obtained by intersecting the differentially expressed genes with the following GO terms: GO:0098609 for cell-cell adhesion and GO:0006836 for neurotransmitter transport.

### Subclustering of neuronal populations

To investigate the diversity of neuronal subtypes, the neuronal population was subdivided into 12 DV domains (see above). Because only a few dI6 neurons were recovered (95 out of 16450 neurons), we excluded this population for the following subclustering analysis. For each of the remaining 11 domains, we pooled together the associated neuronal cells and applied the procedure below:

#### Unbiased identification of transcriptomic features

We reasoned that relevant transcriptomic features involve interacting genes demonstrating concerted patterns of expression. From the initial set of ∼5000 expressed genes, we selected the genes that showed highest Spearman correlation with at least three other genes, lowering the correlation cutoff until ∼2000 genes were retained. These genes were then grouped and further filtered with the following three-step iterative method: (1) The remaining genes were grouped into gene modules by performing a hierarchical clustering using the Spearman dissimilarity matrix of the UMI counts and Ward's agglomeration criterion. The number of modules were set heuristically to 200 to produce compact and homogeneous groupings. (2) A first filtering criterion was applied to test whether enough cells were expressing the genes that comprised the gene module. For each cell, we obtained an average expression level per module by averaging the *z*-scored log-transformed expression levels of all genes that belonged to the module. These gene module averaged levels were then binarised independently using a parameter-free adaptive thresholding method [R function ‘binarize.array()’ from the ArrayBin package]. A cell was considered to express a gene module if the associated Boolean value was true. Modules which were expressed in fewer than five cells were excluded. (3) A second filtering criterion was then applied to test whether cells were expressing the gene module with consistently high levels over most of the genes in the module. We binarised the *z*-scored log-transformed expression levels of all the remaining genes independently. Then, for each module, we calculated the ratio of Boolean values in cells that expressed the module (as defined in 2). We excluded modules in which less than 40% of these Boolean values were true.

The iterative loop was terminated when the number of gene modules converged, i.e. when no gene module was excluded in the last iteration. A summary that indicates the number of expressed genes, correlated genes, unbiasedly identified genes and gene modules per domain is available in Table S4.

#### Curated selection of the relevant features and neuron type clustering

The gene modules were carefully scrutinised using a list of known neuronal marker genes. We isolated a list of curated gene modules that are related to neuronal identities (Table S2 and gene module counts in Table S4). In each domain independently, a variable number of identities were obtained by performing a hierarchical clustering of the cells, using Euclidean distances between *z*-scored log-transformed expression levels of the remaining genes.

### Neuronal trajectories and Pseudotime reconstruction

To project the whole neural dataset into a space that reveals cell-state transitions during neurogenesis and progenitor maturation, we aimed at identifying a set of genes with similar dynamics in each DV domain. In order to reduce any bias toward overrepresented populations, we used a resampled dataset that contained approximately the same number of cells per timepoint and DV domain. The unbiased gene module identification pipeline described above was used to identify 200 gene modules of concerted patterns of expression in the resampled dataset. Among them, four modules were retained as they comprised, respectively, the pan-progenitor marker Sox2, the pan-neuronal marker Tubb3, the early progenitor marker Lin28a and the late progenitor marker Fabp7 (114 genes). An extra step was taken to ensure that no DV bias occurred in the remaining genes by testing for differential expression in response to their DV position (Monocle 2.6.4, ‘differentialGeneTest’ function, *P*<5e-15). Fourteen genes were excluded and 100 retained (Fig. S6A). After taking the log of the normalised UMI counts (‘median ratio’ normalisation method), PCA was then applied to the 100-dimensional resampled dataset. The resulting PC1-PC2 plane was then populated by multiplying the whole (log-normalised) neural dataset by the eigenvector matrix. Neurogenic pseudotime orderings of each cell were mapped to the PC1 coordinates.

Finally, we reconstructed, independently for each domain, smoothed profiles of gene expression along pseudotime by fitting spline curves (Monocle 2.6.4, ‘genSmoothCurve’ with three degrees of freedom). Each profile that was obtained with less than 20 expressed cells was set to zero.

## Supplementary Material

Supplementary information

## References

[DEV173807C1] Alaynick W. A., Jessell T. M. and Pfaff S. L. (2011). SnapShot: spinal cord development. *Cell* 146, 178.e1 10.1016/j.cell.2011.06.038 21729788 PMC3158655

[DEV173807C2] Bikoff J. B., Gabitto M. I., Rivard A. F., Drobac E., Machado T. A., Miri A., Brenner-Morton S., Famojure E., Diaz C., Alvarez F. J. et al. (2016). Spinal inhibitory interneuron diversity delineates variant motor microcircuits. *Cell* 165, 207-219. 10.1016/j.cell.2016.01.027 26949184 PMC4808435

[DEV173807C3] Borowska J., Jones C. T., Zhang H., Blacklaws J., Goulding M. and Zhang Y. (2013). Functional subpopulations of V3 interneurons in the mature mouse spinal cord. *J. Neurosci.* 33, 18553-18565. 10.1523/JNEUROSCI.2005-13.2013 24259577 PMC3894417

[DEV173807C4] Briggs J. A., Li V. C., Lee S., Woolf C. J., Klein A. and Kirschner M. W. (2017). Mouse embryonic stem cells can differentiate via multiple paths to the same state. *eLife* 6, e26945 10.7554/eLife.26945 28990928 PMC5648529

[DEV173807C5] Briscoe J. and Small S. (2015). Morphogen rules: design principles of gradient-mediated embryo patterning. *Development* 142, 3996-4009. 10.1242/dev.129452 26628090 PMC4712844

[DEV173807C6] Carcagno A. L., Di Bella D. J., Goulding M., Guillemot F. and Lanuza G. M. (2014). Neurogenin3 restricts serotonergic neuron differentiation to the hindbrain. *J. Neurosci.* 34, 15223-15233. 10.1523/JNEUROSCI.3403-14.2014 25392491 PMC6608453

[DEV173807C7] Catela C., Shin M. M., Lee D. H., Liu J.-P. and Dasen J. S. (2016). Hox proteins coordinate motor neuron differentiation and connectivity programs through Ret/Gfrα genes. *Cell Rep.* 14, 1901-1915. 10.1016/j.celrep.2016.01.067 26904955 PMC4775310

[DEV173807C9] Dasen J. S., Tice B. C., Brenner-Morton S. and Jessell T. M. (2005). A Hox regulatory network establishes motor neuron pool identity and target-muscle connectivity. *Cell* 123, 477-491. 10.1016/j.cell.2005.09.009 16269338

[DEV173807C10] Dasen J. S., De Camilli A., Wang B., Tucker P. W. and Jessell T. M. (2008). Hox repertoires for motor neuron diversity and connectivity gated by a single accessory factor, FoxP1. *Cell* 134, 304-316. 10.1016/j.cell.2008.06.019 18662545

[DEV173807C11] Demireva E. Y., Shapiro L. S., Jessell T. M. and Zampieri N. (2011). Motor neuron position and topographic order imposed by β- And γ-catenin activities. *Cell* 147, 641-652. 10.1016/j.cell.2011.09.037 22036570 PMC3226777

[DEV173807C12] Deneen B., Ho R., Lukaszewicz A., Hochstim C. J., Gronostajski R. M. and Anderson D. J. (2006). The transcription factor NFIA controls the onset of gliogenesis in the developing spinal cord. *Neuron* 52, 953-968. 10.1016/j.neuron.2006.11.019 17178400

[DEV173807C13] Dias J. M., Alekseenko Z., Applequist J. M. and Ericson J. (2014). Tgfβ signaling regulates temporal neurogenesis and potency of neural stem cells in the CNS. *Neuron* 84, 927-939. 10.1016/j.neuron.2014.10.033 25467979

[DEV173807C14] Dougherty K. J., Zagoraiou L., Satoh D., Rozani I., Doobar S., Arber S., Jessell T. M. and Kiehn O. (2013). Locomotor rhythm generation linked to the output of spinal Shox2 excitatory interneurons. *Neuron* 80, 920-933. 10.1016/j.neuron.2013.08.015 24267650

[DEV173807C15] Ericson J., Thor S., Edlund T., Jessell T. M. and Yamada T. (1992). Early stages of motor neuron differentiation revealed by expression of homeobox gene Islet-1. *Science* 256, 1555-1560. 10.1126/science.1350865 1350865

[DEV173807C16] Ericson J., Morton S., Kawakami A., Roelink H. and Jessell T. M. (1996). Two critical periods of Sonic Hedgehog signaling required for the specification of motor neuron identity. *Cell* 87, 661-673. 10.1016/S0092-8674(00)81386-0 8929535

[DEV173807C17] Francius C. and Clotman F. (2014). Generating spinal motor neuron diversity: a long quest for neuronal identity. *Cell. Mol. Life Sci.* 71, 813-829. 10.1007/s00018-013-1398-x 23765105 PMC11113339

[DEV173807C18] Francius C., Harris A., Rucchin V., Hendricks T. J., Stam F. J., Barber M., Kurek D., Grosveld F. G., Pierani A., Goulding M. et al. (2013). Identification of multiple subsets of ventral interneurons and differential distribution along the rostrocaudal axis of the developing spinal cord. *PLoS ONE* 8, e70325 10.1371/journal.pone.0070325 23967072 PMC3744532

[DEV173807C19] Gabitto M. I., Pakman A., Bikoff J. B., Abbott L. F., Jessell T. M. and Paninski L. (2016). Bayesian sparse regression analysis documents the diversity of spinal inhibitory interneurons. *Cell* 165, 220-233. 10.1016/j.cell.2016.01.026 26949187 PMC4831714

[DEV173807C20] Glasgow S. M., Carlson J. C., Zhu W., Chaboub L. S., Kang P., Lee H. K., Clovis Y. M., Lozzi B. E., McEvilly R. J., Rosenfeld M. G. et al. (2017). Glia-specific enhancers and chromatin structure regulate NFIA expression and glioma tumorigenesis. *Nat. Neurosci.* 20, 1520-1528. 10.1038/nn.4638 28892058 PMC5919190

[DEV173807C21] Gonzalez-Quevedo R., Lee Y., Poss K. D. and Wilkinson D. G. (2010). Neuronal regulation of the spatial patterning of neurogenesis. *Dev. Cell* 18, 136-147. 10.1016/j.devcel.2009.11.010 20152184 PMC2822724

[DEV173807C22] Goulding M. (2009). Circuits controlling vertebrate locomotion: moving in a new direction. *Nat. Rev. Neurosci.* 10, 507-518. 10.1038/nrn2608 19543221 PMC2847453

[DEV173807C23] Gross M. K., Dottori M. and Goulding M. (2002). Lbx1 specifies somatosensory association interneurons in the dorsal spinal cord. *Neuron* 34, 535-549. 10.1016/S0896-6273(02)00690-6 12062038

[DEV173807C24] Hanley O., Zewdu R., Cohen L. J., Jung H., Lacombe J., Philippidou P., Lee D. H., Selleri L. and Dasen J. S. (2016). Parallel Pbx-dependent pathways govern the coalescence and fate of motor columns. *Neuron* 91, 1005-1020. 10.1016/j.neuron.2016.07.043 27568519 PMC5017921

[DEV173807C25] Häring M., Zeisel A., Hochgerner H., Rinwa P., Jakobsson J. E. T., Lönnerberg P., La Manno G., Sharma N., Borgius L., Kiehn O. et al. (2018). Neuronal atlas of the dorsal horn defines its architecture and links sensory input to transcriptional cell types. *Nat. Neurosci.* 21, 869-880. 10.1038/s41593-018-0141-1 29686262

[DEV173807C26] Harris A., Masgutova G., Collin A., Toch M., Hidalgo-Figueroa M., Jacob B., Corcoran L. M., Francius C., Clotman F. (2018). Onecut factors and Pou2f2 regulate diversification and migration of V2 interneurons in the mouse developing spinal cord *bioRxiv* 10.1101/413054. PMC6561314 31231191

[DEV173807C27] Hayashi M., Hinckley C. A., Driscoll S. P., Moore N. J., Levine A. J., Hilde K. L., Sharma K. and Pfaff S. L. (2018). Graded arrays of spinal and supraspinal V2a interneuron subtypes underlie forelimb and hindlimb motor control. *Neuron* 97, 869-884. 10.1016/j.neuron.2018.01.023 29398364 PMC8601153

[DEV173807C28] Hoang P. T., Chalif J. I., Bikoff J. B., Jessell T. M., Mentis G. Z. and Wichterle H. (2018). Subtype diversification and synaptic specificity of stem cell-derived spinal interneurons. *Neuron* 100, 135-149.e7. 10.1016/j.neuron.2018.09.016 30308166 PMC6590086

[DEV173807C29] Holguera I. and Desplan C. (2018). Neuronal specification in space and time. *Science* 362, 176-180. 10.1126/science.aas9435 30309944 PMC6368964

[DEV173807C30] Hollyday M. and Hamburger V. (1977). An autoradiographic study of the formation of the lateral motor column in the chick embryo. *Brain Res.* 132, 197-208. 10.1016/0006-8993(77)90416-4 890480

[DEV173807C31] Jessell T. M. (2000). Neuronal specification in the spinal cord: inductive signals and transcriptional codes. *Nat. Rev. Genet.* 1, 20-29. 10.1038/35049541 11262869

[DEV173807C32] Kang P., Lee H. K., Glasgow S. M., Finley M., Donti T., Gaber Z. B., Graham B. H., Foster A. E., Novitch B. G., Gronostajski R. M. et al. (2012). Sox9 and NFIA coordinate a transcriptional regulatory cascade during the initiation of gliogenesis. *Neuron* 74, 79-94. 10.1016/j.neuron.2012.01.024 22500632 PMC3543821

[DEV173807C33] Kicheva A. and Briscoe J. (2015). Developmental pattern formation in phases. *Trends Cell Biol.* 25, 579-591. 10.1016/j.tcb.2015.07.006 26410404

[DEV173807C34] Kicheva A., Bollenbach T., Ribeiro A., Valle H. P., Lovell-Badge R., Episkopou V. and Briscoe J. (2014). Coordination of progenitor specification and growth in mouse and chick spinal cord. *Science* 345, 1254927 10.1126/science.1254927 25258086 PMC4228193

[DEV173807C35] Kiehn O. (2016). Decoding the organization of spinal circuits that control locomotion. *Nat. Rev. Neurosci.* 17, 224-238. 10.1038/nrn.2016.9 26935168 PMC4844028

[DEV173807C36] Kutejova E., Sasai N., Shah A., Gouti M. and Briscoe J. (2016). Neural progenitors adopt specific identities by directly repressing all alternative progenitor transcriptional programs. *Dev. Cell* 36, 639-653. 10.1016/j.devcel.2016.02.013 26972603 PMC4819439

[DEV173807C37] Lai H. C., Seal R. P. and Johnson J. E. (2016). Making sense out of spinal cord somatosensory development. *Development* 143, 3434-3448. 10.1242/dev.139592 27702783 PMC5087618

[DEV173807C38] Landmesser L. T. (2001). The acquisition of motoneuron subtype identity and motor circuit formation. *Int. J. Dev. Neurosci.* 19, 175-182. 10.1016/S0736-5748(00)00090-3 11255031

[DEV173807C39] Le Dréau G. and Martí E. (2012). Dorsal-ventral patterning of the neural tube: a tale of three signals. *Dev. Neurobiol.* 72, 1471-1481. 10.1002/dneu.22015 22821665

[DEV173807C40] Lee S.-K. and Pfaff S. L. (2001). Transcriptional networks regulating neuronal identity in the developing spinal cord. *Nat. Neurosci.* 4 Suppl., 1183-1191. 10.1038/nn750 11687828

[DEV173807C41] Lin J., Wang C. and Redies C. (2012). Expression of delta-protocadherins in the spinal cord of the chicken embryo. *J. Comp. Neurol.* 520, 1509-1531. 10.1002/cne.22808 22102158

[DEV173807C42] Lu D. C., Niu T. and Alaynick W. A. (2015). Molecular and cellular development of spinal cord locomotor circuitry. *Front. Mol. Neurosci.* 8, 25 10.3389/fnmol.2015.00025 26136656 PMC4468382

[DEV173807C43] Lutz B., Kuratani S., Cooney A. J., Wawersik S., Tsai S. Y., Eichele G. and Tsai M. J. (1994). Developmental regulation of the orphan receptor COUP-TF II gene in spinal motor neurons. *Development* 120, 25-36. 8119130 10.1242/dev.120.1.25

[DEV173807C44] Morikawa Y., Hisaoka T. and Senba E. (2009). Characterization of Foxp2-expressing cells in the developing spinal cord. *Neuroscience* 162, 1150-1162. 10.1016/j.neuroscience.2009.05.022 19463901

[DEV173807C45] Morita K., Furuse M., Fujimoto K. and Tsukita S. (1999). Claudin multigene family encoding four-transmembrane domain protein components of tight junction strands. *Proc. Natl. Acad. Sci. USA* 96, 511-516. 10.1073/pnas.96.2.511 9892664 PMC15167

[DEV173807C46] Müller T., Brohmann H., Pierani A., Heppenstall P. A., Lewin G. R., Jessell T. M. and Birchmeier C. (2002). The homeodomain factor Lbx1 distinguishes two major programs of neuronal differentiation in the dorsal spinal cord. *Neuron* 34, 551-562. 10.1016/S0896-6273(02)00689-X 12062039

[DEV173807C47] Müller T., Anlag K., Wildner H., Britsch S., Treier M. and Birchmeier C. (2005). The bHLH factor Olig3 coordinates the specification of dorsal neurons in the spinal cord. *Genes Dev.* 19, 733-743. 10.1101/gad.326105 15769945 PMC1065726

[DEV173807C48] Niederreither K., McCaffery P., Dräger U. C., Chambon P. and Dollé P. (1997). Restricted expression and retinoic acid-induced downregulation of the retinaldehyde dehydrogenase type 2 (RALDH-2) gene during mouse development. *Mech. Dev.* 62, 67-78. 10.1016/S0925-4773(96)00653-3 9106168

[DEV173807C49] Nishi Y., Zhang X., Jeong J., Peterson K. A., Vedenko A., Bulyk M. L., Hide W. A. and McMahon A. P. (2015). A direct fate exclusion mechanism by Sonic hedgehog-regulated transcriptional repressors. *Development* 142, 3286-3293. 10.1242/dev.124636 26293298 PMC4631756

[DEV173807C50] Novitch B. G., Chen A. I. and Jessell T. M. (2001). Coordinate regulation of motor neuron subtype identity and pan-neuronal properties by the bHLH repressor Olig2. *Neuron* 31, 773-789. 10.1016/S0896-6273(01)00407-X 11567616

[DEV173807C51] Novitch B. G., Wichterle H., Jessell T. M. and Sockanathan S. (2003). A requirement for retinoic acid-mediated transcriptional activation in ventral neural patterning and motor neuron specification. *Neuron* 40, 81-95. 10.1016/j.neuron.2003.08.006 14527435

[DEV173807C52] Philippidou P. and Dasen J. S. (2013). Hox genes: choreographers in neural development, architects of circuit organization. *Neuron* 80, 12-34. 10.1016/j.neuron.2013.09.020 24094100 PMC3835187

[DEV173807C53] Preibisch S., Saalfeld S. and Tomancak P. (2009). Globally optimal stitching of tiled 3D microscopic image acquisitions. *Bioinformatics* 25, 1463-1465. 10.1093/bioinformatics/btp184 19346324 PMC2682522

[DEV173807C54] Price S. R., De Marco Garcia N. V., Ranscht B. and Jessell T. M. (2002). Regulation of motor neuron pool sorting by differential expression of type II cadherins. *Cell* 109, 205-216. 10.1016/S0092-8674(02)00695-5 12007407

[DEV173807C55] Rosenberg A. B., Roco C. M., Muscat R. A., Kuchina A., Sample P., Yao Z., Graybuck L. T., Peeler D. J., Mukherjee S., Chen W. et al. (2018). Single-cell profiling of the developing mouse brain and spinal cord with split-pool barcoding. *Science* 360, 176-182. 10.1126/science.aam8999 29545511 PMC7643870

[DEV173807C56] Rousso D. L., Gaber Z. B., Wellik D., Morrisey E. E. and Novitch B. G. (2008). Coordinated actions of the Forkhead protein Foxp1 and Hox proteins in the columnar organization of spinal motor neurons. *Neuron* 59, 226-240. 10.1016/j.neuron.2008.06.025 18667151 PMC2547125

[DEV173807C57] Roy A., Francius C., Rousso D. L., Seuntjens E., Debruyn J., Luxenhofer G., Huber A. B., Huylebroeck D., Novitch B. G. and Clotman F. (2012). Onecut transcription factors act upstream of Isl1 to regulate spinal motoneuron diversification. *Development* 139, 3109-3119. 10.1242/dev.078501 22833130 PMC4074251

[DEV173807C58] Sagner A., Gaber Z. B., Delile J., Kong J. H., Rousso D. L., Pearson C. A., Weicksel S. E., Melchionda M., Mousavy Gharavy S. N., Briscoe J. et al. (2018). Olig2 and Hes regulatory dynamics during motor neuron differentiation revealed by single cell transcriptomics. *PLoS Biol.* 16, e2003127 10.1371/journal.pbio.2003127 29389974 PMC5811045

[DEV173807C59] Sathyamurthy A., Johnson K. R., Matson K. J. E., Dobrott C. I., Li L., Ryba A. R., Bergman T. B., Kelly M. C., Kelley M. W. and Levine A. J. (2018). Massively parallel single nucleus transcriptional profiling defines spinal cord neurons and their activity during behavior. *Cell Rep.* 22, 2216-2225. 10.1016/j.celrep.2018.02.003 29466745 PMC5849084

[DEV173807C60] Saunders A., Macosko E. Z., Wysoker A., Goldman M., Krienen F. M., de Rivera H., Bien E., Baum M., Bortolin L., Wang S. et al. (2018). Molecular diversity and specializations among the cells of the adult mouse brain. *Cell* 174, 1015-1030.e16. 10.1016/j.cell.2018.07.028 30096299 PMC6447408

[DEV173807C61] Setty M., Tadmor M. D., Reich-zeliger S., Angel O., Salame T. M., Kathail P., Choi K., Bendall S., Friedman N. and Pe D. (2016). Articles Wishbone identifies bifurcating developmental trajectories from single-cell data. *Nat. Biotechnol.* 34, 614-637. 10.1038/nbt.3569 PMC4900897 27136076

[DEV173807C62] Sharma K., Sheng H. Z., Lettieri K., Li H., Karavanov A., Potter S., Westphal H. and Pfaff S. L. (1998). LIM homeodomain factors Lhx3 and Lhx4 assign subtype identities for motor neurons. *Cell* 95, 817-828. 10.1016/S0092-8674(00)81704-3 9865699

[DEV173807C63] Shekhar K., Lapan S. W., Whitney I. E., Tran N. M., Macosko E. Z., Kowalczyk M., Adiconis X., Levin J. Z., Nemesh J., Goldman M. et al. (2016). Comprehensive classification of retinal bipolar neurons by single-cell transcriptomics. *Cell* 166, 1308-1323.e30. 10.1016/j.cell.2016.07.054 27565351 PMC5003425

[DEV173807C64] Shin J., Berg D. A., Christian K. M., Shin J., Berg D. A., Zhu Y., Shin J. Y., Song J. and Bonaguidi M. A. (2015). Single-cell RNA-Seq with waterfall reveals molecular cascades underlying adult neurogenesis resource single-cell RNA-Seq with waterfall reveals molecular cascades underlying adult neurogenesis. *Stem Cell* 17, 360-372. 10.1016/j.stem.2015.07.013 PMC8638014 26299571

[DEV173807C65] Sockanathan S. and Jessell T. M. (1998). Motor neuron–derived retinoid signaling specifies the subtype identity of spinal motor neurons. *Cell* 94, 503-514. 10.1016/S0092-8674(00)81591-3 9727493

[DEV173807C66] Sommer L., Ma Q. and Anderson D. J. (1996). neurogenins, a novel family of atonal-related bHLH transcription factors, are putative mammalian neuronal determination genes that reveal progenitor cell heterogeneity in the developing CNS and PNS. *Mol. Cell. Neurosci.* 8, 221-241. 10.1006/mcne.1996.0060 9000438

[DEV173807C67] Stam F. J., Hendricks T. J., Zhang J., Geiman E. J., Francius C., Labosky P. A., Clotman F. and Goulding M. (2012). Renshaw cell interneuron specialization is controlled by a temporally restricted transcription factor program. *Development* 139, 179-190. 10.1242/dev.071134 22115757 PMC3231776

[DEV173807C68] Stifani N. (2014). Motor neurons and the generation of spinal motor neuron diversity. *Front. Cell. Neurosci.* 8, 293 10.3389/fncel.2014.00293 25346659 PMC4191298

[DEV173807C69] Stolt C. C., Lommes P., Sock E., Chaboissier M. C., Schedl A. and Wegner M. (2003). The Sox9 transcription factor determines glial fate choice in the developing spinal cord. *Genes Dev.* 17, 1677-1689. 10.1101/gad.259003 12842915 PMC196138

[DEV173807C70] Südhof T. C. (2017). Synaptic neurexin complexes: a molecular code for the logic of neural circuits. *Cell* 171, 745-769. 10.1016/j.cell.2017.10.024 29100073 PMC5694349

[DEV173807C71] Sweeney L. B., Bikoff J. B., Gabitto M. I., Brenner-Morton S., Baek M., Yang J. H., Tabak E. G., Dasen J. S., Kintner C. R. and Jessell T. M. (2018). Origin and segmental diversity of spinal inhibitory interneurons. *Neuron* 97, 341-355.e3. 10.1016/j.neuron.2017.12.029 29307712 PMC5880537

[DEV173807C73] Trapnell C., Cacchiarelli D., Grimsby J., Pokharel P., Li S., Morse M., Lennon N. J., Livak K. J., Mikkelsen T. S. and Rinn J. L. (2014). The dynamics and regulators of cell fate decisions are revealed by pseudotemporal ordering of single cells. *Nat. Biotechnol.* 32, 381-386. 10.1038/nbt.2859 24658644 PMC4122333

[DEV173807C74] Tripodi M., Stepien A. E. and Arber S. (2011). Motor antagonism exposed by spatial segregation and timing of neurogenesis. *Nature* 479, 61-66. 10.1038/nature10538 22012263

[DEV173807C75] Uemura T., Lee S.-J., Yasumura M., Takeuchi T., Yoshida T., Ra M., Taguchi R., Sakimura K. and Mishina M. (2010). Trans-synaptic interaction of GluRδ2 and neurexin through Cbln1 mediates synapse formation in the cerebellum. *Cell* 141, 1068-1079. 10.1016/j.cell.2010.04.035 20537373

[DEV173807C77] Zeisel A., Hochgerner H., Lönnerberg P., Johnsson A., Memic F., van der Zwan J., Häring M., Braun E., Borm L. E., La Manno G. et al. (2018). Molecular architecture of the mouse nervous system. *Cell* 174, 999-1014.e22. 10.1016/j.cell.2018.06.021 30096314 PMC6086934

