# Supplementary material for: Single cell transcriptomics reveals spatial and temporal dynamics of gene expression in the developing mouse spinal cord
Source: Development. 2019 Mar 27;146(12):dev173807. doi: 10.1242/dev.173807 (PMC6602353; doi:10.1242/dev.173807)
Supplement: Supplementary information [file develop-146-173807-s1.pdf]

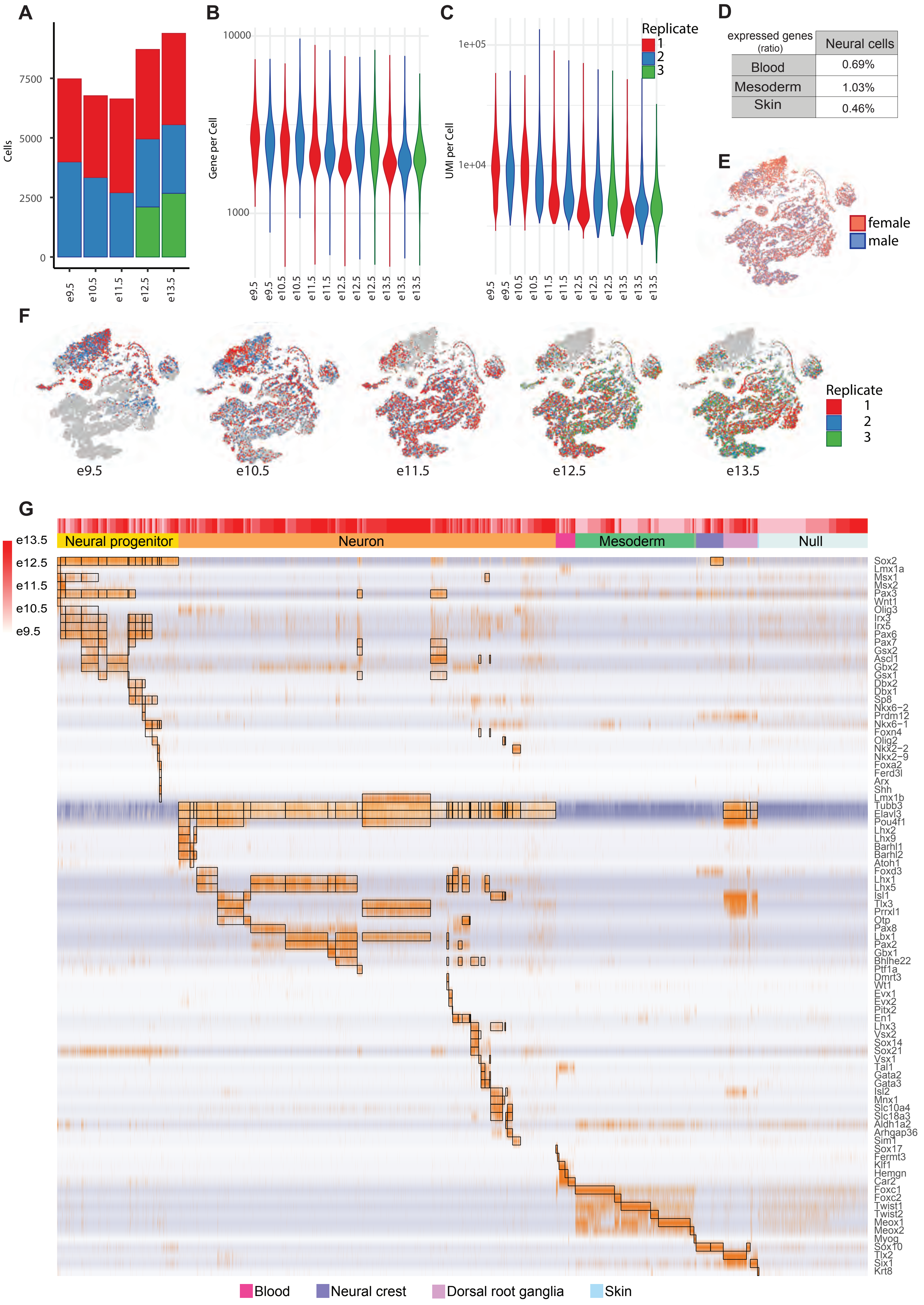

Development • Supplementary information

Figure S1: Further characterization of the single cell transcriptome dataset.
(A) Number of cells per replicate and timepoint.
(B) Number of genes per cell for each replicate.
(C) Number of unique molecular identifiers (UMIs) per cell for each replicate.
(D) Ratio of blood (Sox17, Fermt3, Klf1, Hemgn, Car2), mesoderm (Foxc1, Foxc2, Twist1, Twist2, Meox1, Meox2) and skin (Krt8) markers expressed in neural cells.
(E,F) tSNE-plots showing the distribution of male and female cells in the dataset (E), and the different replicates per timepoint (F).
(G) Heatmap showing read distributions of the genes used to assign cell identities to the single cell transcriptomic profiles. Boxed regions indicate the cell identities in which the respective gene is expected to be expressed. Bars at the top of the heatmap indicate sample age (grey early, late red), and cell identity as color coded in Fig 1.

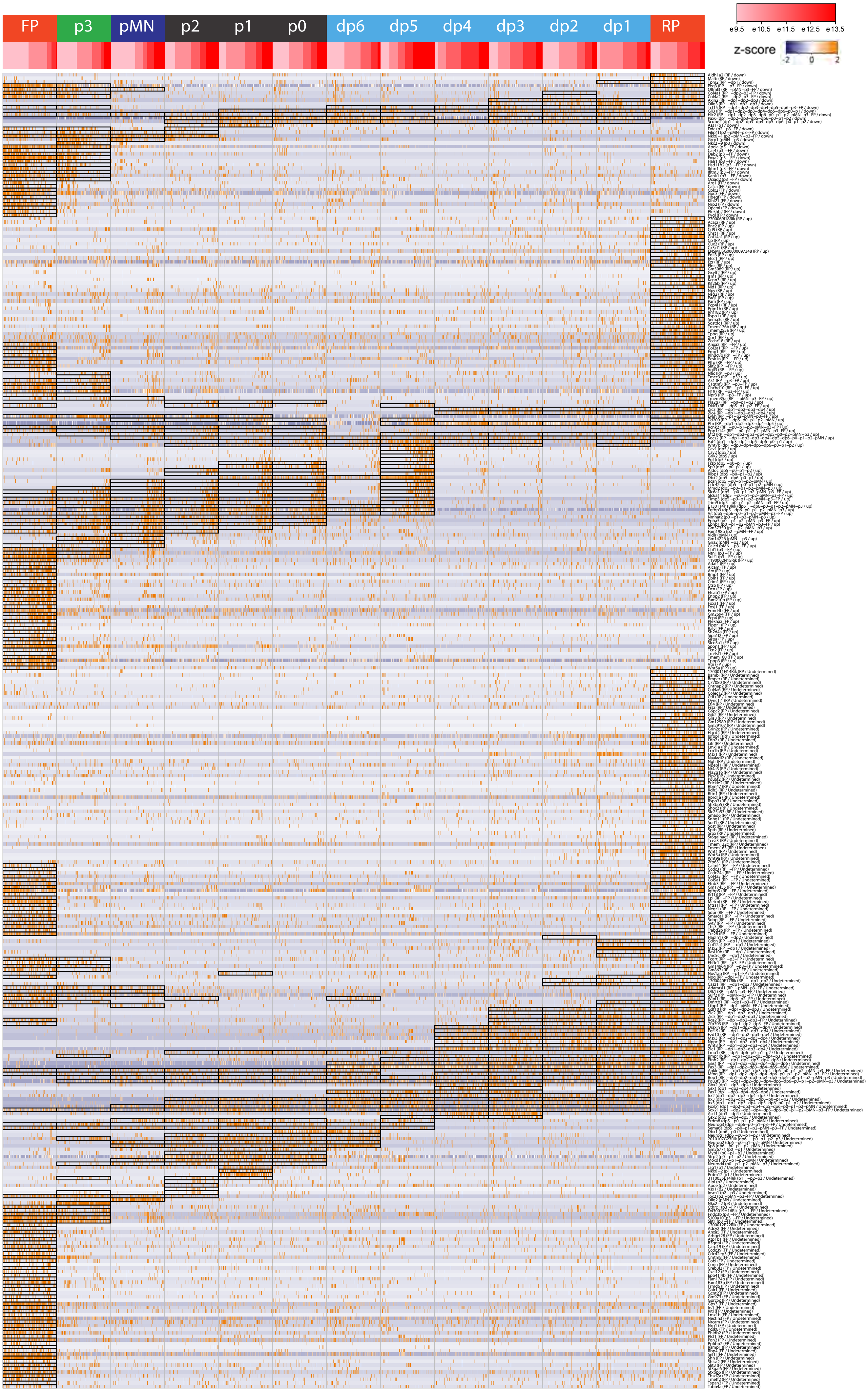
FP
p3
pMN
p2
p1
p0
dp6
dp5
dp4
dp3
dp2
dp1
RP
e9.5
e10.5
e11.5
e12.5
e13.5
z-score
-2
0
2

Figure S2: Differentially expressed genes between the different neural progenitor domains. Heatmap showing z-scored UMI count distributions of the genes identified as differentially expressed. Domains predicted to express a gene are indicated by boxes. Cells within each domain are ordered by sample time. Rows are ordered according to the correlation between gene expression and sample time (first downregulated genes, $r < -.2$, then upregulated genes, $r > .2$, and finally undetermined trends).
.

Development • Supplementary information

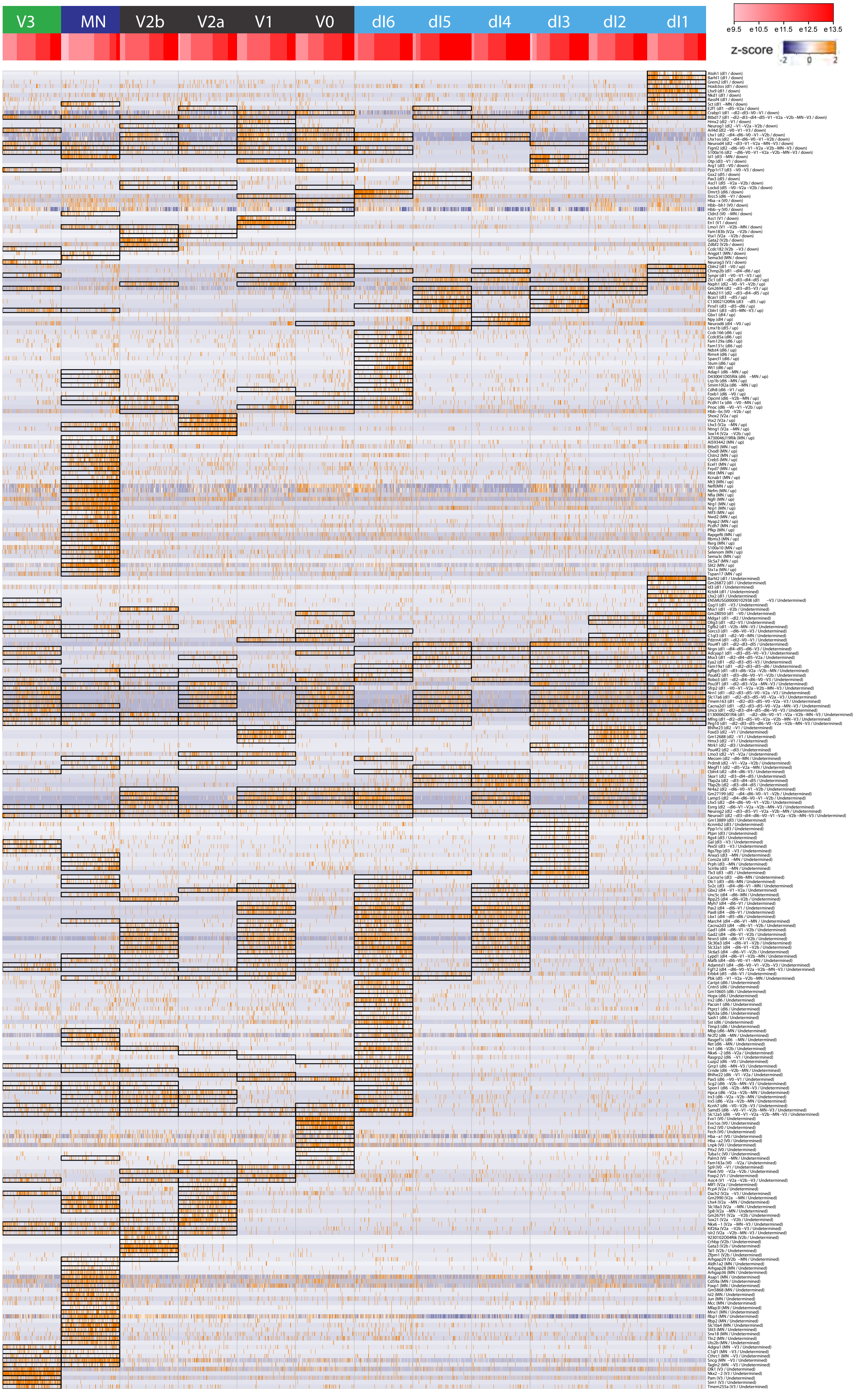

Development • Supplementary information

Figure S3: Differentially expressed genes between the different neuronal domains.
Heatmap showing z-scored UMI count distributions of the genes identified as differentially expressed. Domains predicted to express a gene are indicated by boxes. Cells within each domain are ordered by sample time. Rows are ordered according to the correlation between gene expression and sample time (first downregulated genes, $r < -.2$, then upregulated genes, $r > .2$, and finally indeterminant trends).

.

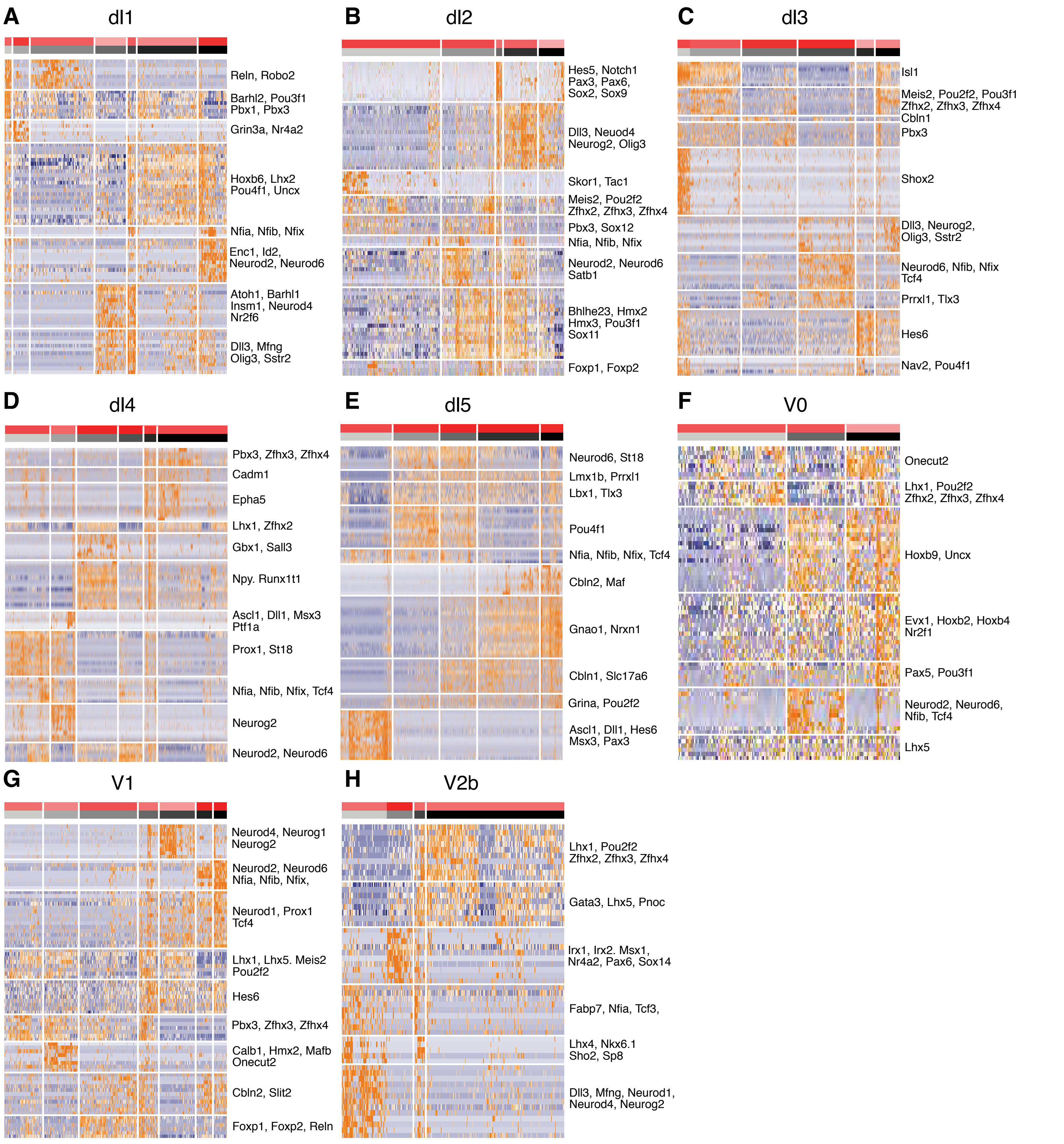

Figure S4: Hierarchical clustering of dI1, dI2, dI3, dI4, dI5, V0, V1 and V2b neurons.
Characterization of neuronal subtypes by hierarchical clustering of the indicated domains. Hierarchical clustering was performed using the curated gene modules available in Table S2. A subset of genes included in the modules are indicated on the righthand side.

Development • Supplementary information

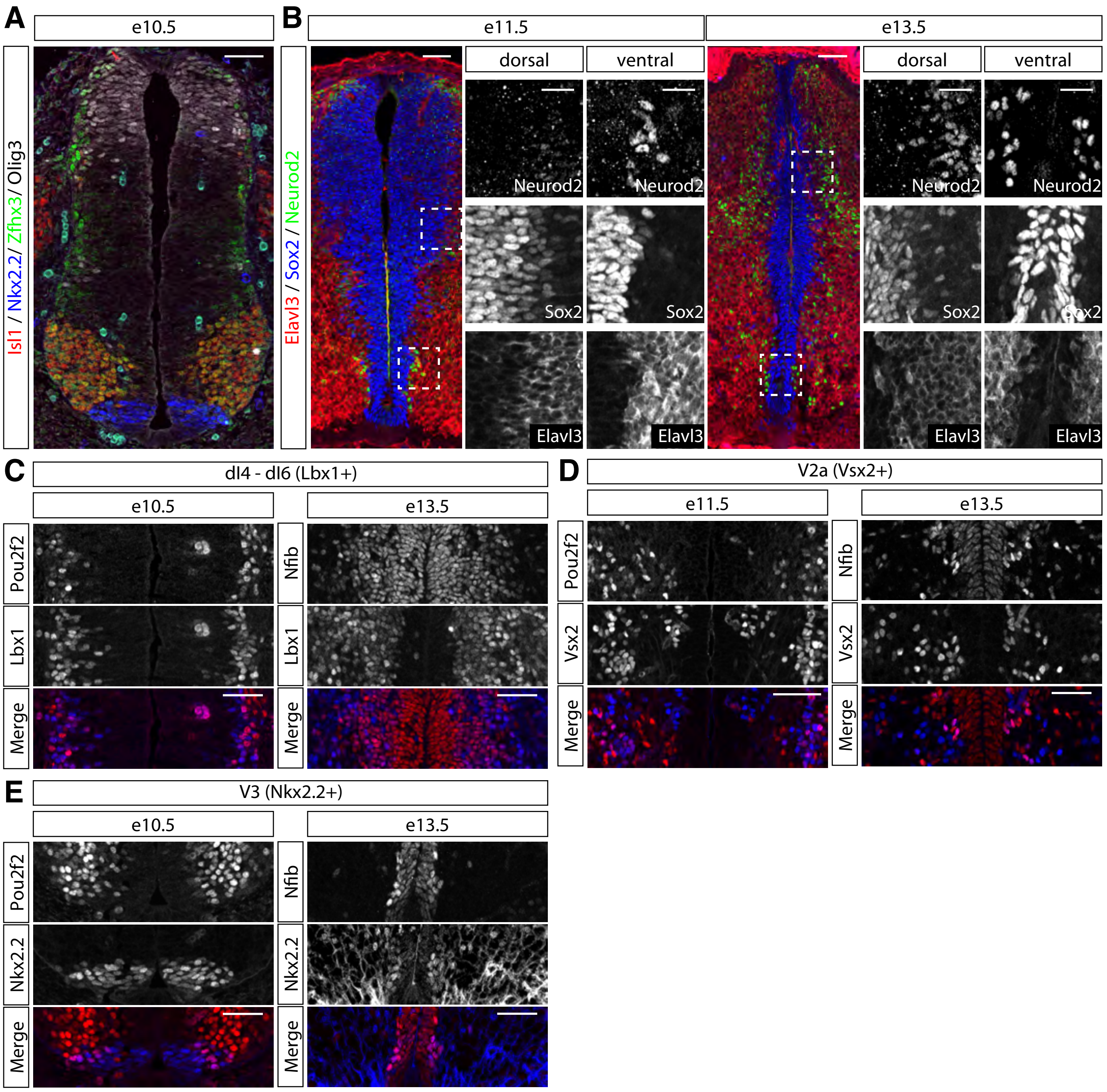

Figure S5: Temporal stratification of neuronal subtypes by a shared set of TFs

(A) Expression of Zfhx3 in neurons at e10.5

(B) Increased expression of Neurod2 in neurons after e11.5. At e11.5 only few neurons in the very dorsal and ventral spinal cord express Neurod2. Number of Neurod2 positive neurons strongly increases between e11.5 and e13.5.

(C-E) Coexpression of Pou2f2 with specific markers for multiple domains at early developmental stages (e10.5 for Nkx2.2 and Lbx1, e11.5 for Vsx2). At e13.5, Nfib is expressed in the same domains in a subset of neurons. These neurons are typically adjacent to the ventricular zone, consistent with their late birth date.

Scale bars = 50 μm, scale bars of insets are 25 μm

.

Development • Supplementary information

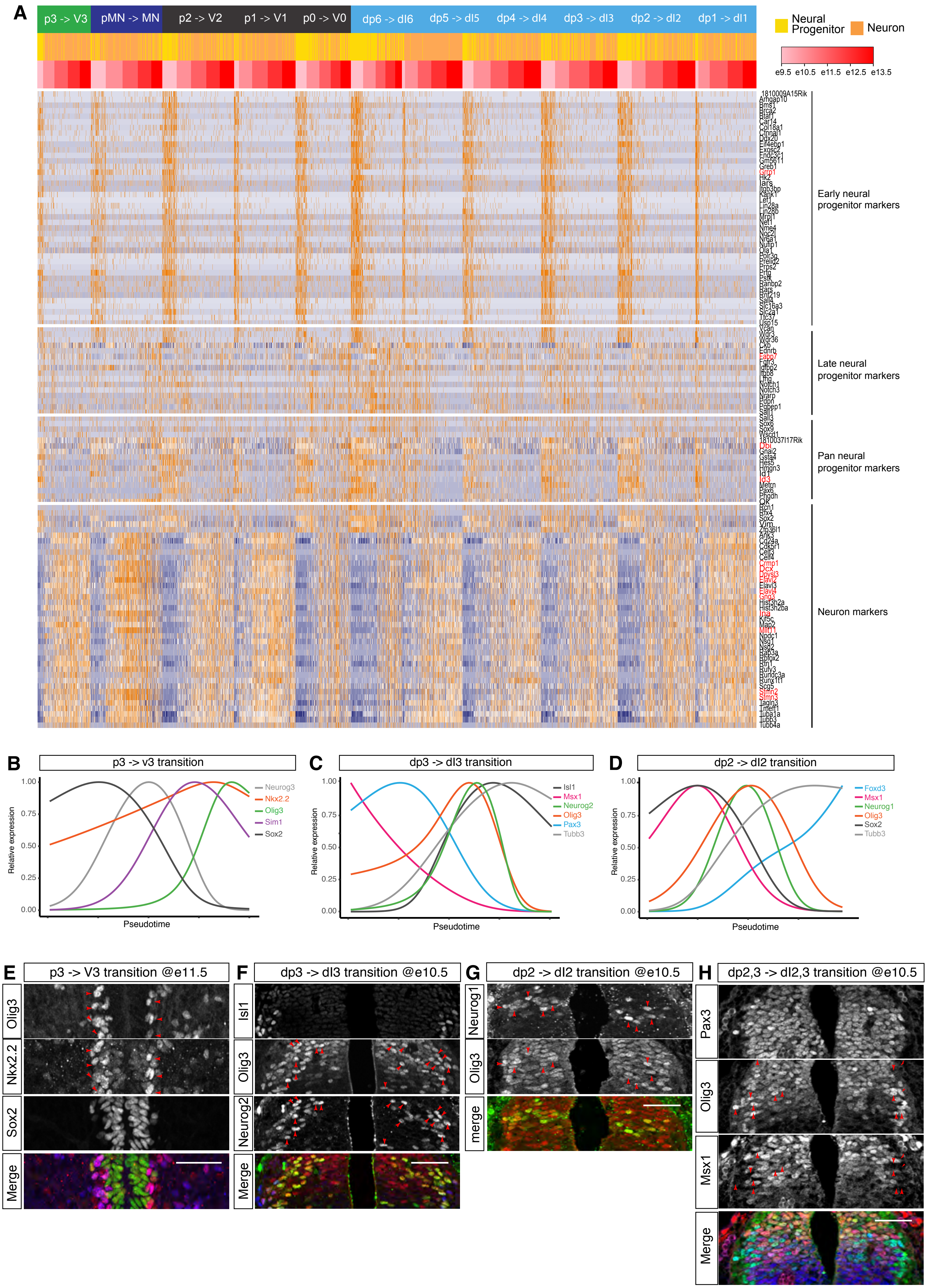

Development • Supplementary information

Figure S6: Pseudotime analysis reveals gene expression dynamics underlying neurogenesis in several domains.
(A) Heatmap showing the 4 genes modules identified as similarly expressed in all dorsal-ventral domains and involved in neurogenesis and progenitor maturation. Among the 114 genes, red gene labels indicate genes excluded from the following analysis because of significant dorsal-ventral bias.
(B) Smoothed expression profile of the neurogenic trajectory from p3 to V3 shows increasing levels of Nkx2.2 during neurogenesis. Note that Nkx2.2 in contrast to other neural progenitor markers continues to be expressed in V3 neurons.
(C) Smoothed expression profile of the neurogenic trajectory from dp3 to dI3 shows a transient upregulation of Olig3 prior to neurogenesis that coincides with the maximal expression of Neurog2.
(D) Smoothed expression profile of the neurogenic trajectory from dp2 to dI2 shows a transient upregulation of Olig3 prior to neurogenesis that coincides with the maximal expression of Neurog1.
(E) Upregulation of Nkx2.2 in Olig3+ (red arrows) V3 neurons compared to Sox2+ Nkx2.2 p3 progenitors at e11.5.
(F) Upregulation of Olig3 in neurogenic dp3 progenitors labelled by Neurog2 (red arrows).
(G) Upregulation of Olig3 in neurogenic dp2 progenitors labelled by Neurog1 (red arrows).
(H) Specific upregulation of Olig3 is anti-correlated with Msx1 (red arrows). The broadly dorsal progenitor marker Pax3 is homogeneously expressed in dp2 and dp3 domains.
Scale bars = 50 μm

Development • Supplementary information

SUPPLEMENTAL TABLES

Table S1. Knowledge matrix used to identify cell types. Columns indicate the cell type classes and rows the genes. A gene contributes to a class definition if the associated value is 1.

Click here to Download Table S1

Table S2. Curated gene lists used to cluster the neuronal subtypes in each dorsal-ventral domain.

Click here to Download Table S2

Table S3. List of primary antibodies including manufacturer and the dilutions used.

Click here to Download Table S3

Table S4. Summary table indicating the number of genes and modules for each step of the gene module identification method used to cluster the neuronal subtypes.

Click here to Download Table S4

Development • Supplementary information
